# The American Transformative HIV Study: Protocol for a US National Cohort of Sexual and Gender Minority Individuals With HIV

**DOI:** 10.2196/66921

**Published:** 2025-05-22

**Authors:** Christian Grov, Alexa B D'Angelo, Chloe Mirzayi, Michelle Dearolf, Elena Hoeppner, Yan Guo, Nicole Richards, Rifa Ehsan, Sarah Kulkarni, Denis Nash, Viraj V Patel, Dustin T Duncan, Meredith Ray, Tyler Bartholomew, Jennifer Manuzak, Jennifer Manuel, Kathryn McCollister, Drew Westmoreland, Adam W Carrico

**Affiliations:** 1 City University of New York Graduate School of Public Health and Health Policy, 55 West 125 Street, New York, NY, 10027, United States, 1 6463640254; 2 Albert Einstein College of Medicine, Montefiore Health System, New York, NY, United States; 3 Columbia University, New York, NY, United States; 4 The University of Memphis, Memphis, TN, United States; 5 University of Miami, Coral Gables, FL, United States; 6 Tulane University, New Orleans, LA, United States; 7 University of California San Francisco, San Francisco, CA, United States; 8 University of Florida, Gainesville, FL, United States; 9 Florida International University, Miami, FL, United States

**Keywords:** HIV testing, rectal cytokines or chemokines, cohort research, nested case-cohort designs, American Transformative HIV Study, AMETHST, United States, sexual and gender minority, methamphetamine, web-based survey, oral fluid, rectal swab

## Abstract

**Background:**

Sexual and gender minority (SGM) individuals represent 2%‐5% of the US population, yet continue to account for more than two-thirds of new HIV infections annually.

**Objective:**

This study seeks to identify multilevel (ie, structural, psychological, and social) and biobehavioral (ie, rectal cytokines or chemokines) determinants of amplified HIV seroconversion risk for SGM individuals, including those who use methamphetamine.

**Methods:**

The American Transformative HIV Study is an ongoing web-based cohort study of 5364 SGM individuals from all 50 US states and Puerto Rico, enrolled in 2022 and 2023, and will be followed through 2027. We oversampled persons who use methamphetamine (2846/5364, 53.1%). We used established web-based strategies to enroll individuals aged 16‐49 years at high risk of HIV acquisition via sexual networking apps. To be eligible, participants had to report meeting objective criteria for HIV pre-exposure prophylaxis (PrEP) care, but not be taking PrEP. Participants complete annual web-based surveys (baseline, 12, 24, and 36 months) and are asked to provide self-collected oral fluid samples for HIV testing and 2 rectal swabs (the Aptima Multitest Swab and the Zymo DNA/RNA Shield swab) following each assessment. Oral fluid samples are analyzed immediately, while rectal swabs are banked for a future nested case-cohort analysis to assess changes in inflammatory markers following a new infection.

**Results:**

Nearly all participants (4542/5364, 84.7%) were cisgender men, 3.7% (201/5364) were transgender women, and 1.1% (61/5364) were transgender men who have sex with men. There were also 560 (10.4%) individuals who self-identified outside of the gender binary—all reported being assigned male sex at birth. In total, 56.5% (3031/5364) were persons of color, and 31.8% (1714/5365) were aged 16 to 29 years. In total, 4054 baseline HIV test kits were returned, including 371 HIV reactive samples—3.3% (69/2210) were HIV-positive among those who did not report methamphetamine use, and 15.5% (302/1944) were HIV-positive among those reporting methamphetamine use. Based on participant’s HIV results as well as self-reporting when their most recent prior HIV-negative test was, we estimated that the incidence rate in this cohort in the 12-month period leading up to study enrollment was 10.06 (95% CI 8.65‐11.64) per 100 person-years among those reporting methamphetamine use compared with 2.04 (95% CI 1.49‐2.73) among those not reporting methamphetamine use per 100 person-years.

**Conclusions:**

A large, US national, and racially diverse web-based cohort of SGM individuals at high risk for HIV has been successfully enrolled and will be followed through 2027. Persons who use methamphetamine have been oversampled and demonstrated an exceptionally greater risk for HIV. Our study will offer insight into the development and implementation of new interventions, which aim to have a meaningful impact on HIV transmission.

## Introduction

### Background

Sexual and gender minority (SGM) individuals represent 2%‐5% of the US population, yet continue to account for more than two-thirds of new HIV infections annually [1-4]. The Centers for Disease Control and Prevention has estimated that 1 in 6 sexual minority men will acquire HIV in their lifetime, including half of Black sexual minority men and one-quarter of Latino sexual minority men [2,5]. Meanwhile, gender minority individuals (transgender men and transgender women) accounted for 2% of new HIV infections in 2019 (while representing ~1% of the US population) [6-8], with transwomen of color most impacted [8]. A Centers for Disease Control and Prevention survey of transgender women across 7 US cities found that 42% of respondents were living with HIV, with 65% of American Indian or Alaska Natives, 62% of Black transwomen, and 35% of Latinx transwomen living with HIV [9]. Our ability to avert new infections may be further challenged by racial disparities in pre-exposure prophylaxis (PrEP) uptake [10].

Methamphetamine use is an enduring and resurgent epidemic in SGM communities [11-15]. Rates of methamphetamine use are high among SGM individuals, ranging from 7.4% to 30%. Although methamphetamine use may have declined somewhat after significant public health attention in the early- and mid-2000s [16,17], it is again on the rise [16,18-22]. Findings from our team and others [23,24] demonstrate that methamphetamine use is disproportionately impacting SGM individuals who are from racial and ethnic minority groups and accounts for one-in-three new HIV infections in SGM individuals [25]. There are behavioral, structural, psychosocial, and biological pathways through which methamphetamine increases the risk for HIV transmission.

### Mechanisms That Increase HIV Vulnerability

#### Behavioral

Methamphetamine increases sexual libido while decreasing inhibitions [26-28]. Because it is a stimulant, sexual minority men using methamphetamine will often engage in condomless anal sex (CAS) with multiple partners for prolonged periods (1‐3 days) [28-30], with less regard to HIV prevention modalities.

#### Structural

High rates of methamphetamine use and its social acceptability in sexual minority men communities potentiate exposure risk through social or sexual networks [31-34]. Many sexual minority men are also highly likely to reside in concentrated neighborhoods within urban centers where the public health impact of methamphetamine use is amplified. Meanwhile, institutionalized and cultural stigma, homophobia, and discrimination affect the health of SGM individuals [35-41], which can keep them from seeking HIV prevention services and substance use disorder treatment [42]. These can include individualized minority stress exposures (eg, being subject to a homophobic slur) and structural determinants (eg, living in an area with unfavorable SGM policies or laws).

#### Psychosocial

There are known psychosocial determinants that increase the risk for methamphetamine use and HIV. Syndemic theory posits that multiple co-occurring epidemics work synergistically to increase—for instance—HIV vulnerability in SGM individuals [43-48]. In syndemic theory, “epidemics” refers both to epidemics of disease [49] (eg, mental health issues, methamphetamine use, and HIV) and social epidemics (eg, poverty, racism, and homophobia). Among SGM individuals—especially SGM persons of color—simultaneous and synergistic epidemics increase HIV vulnerability [43,47,48,50,51]. For example, studies of Black and Latino sexual minority men draw associations among methamphetamine use, mental health outcomes, and condomless sex, all of which could amplify HIV vulnerability [51].

#### Biological

Methamphetamine potentiates rectal immune dysregulation in SGM individuals, which could amplify the biological risk for HIV. Approximately 70% of HIV infections in sexual minority men occur during receptive CAS [52-54], but little is known about the correlates and consequences of rectal immune dysregulation relevant to HIV [55]. Methamphetamine directly decreases parasympathetic tone [56], increasing intestinal permeability and altering rectal immune responses by changing the gut microbiome [57]. Methamphetamine also directly damages gut barrier integrity, which could amplify rectal immune dysregulation [58].

### Study Objective and Hypothesis

The objective of this study is to identify multilevel (ie, structural, psychological, and social) and biological (ie, rectal cytokines or chemokines) determinants of amplified HIV seroconversion risk for SGM individuals who use methamphetamine to guide targeted, combination interventions to reduce HIV incidence. We hypothesize that those reporting methamphetamine use would have higher HIV prevalence as well as higher recent HIV incidence than those not reporting methamphetamine use.

## Methods

### Overview

We sought to recruit, via sexual networking apps, a racially and geographically diverse sample of HIV-negative SGM individuals who are not on PrEP, to better inform the design, implementation, scale-up, and evaluation of HIV prevention programs. Moreover, to understand the multilevel biobehavioral pathways through which methamphetamine amplifies HIV risk, we sought to overenroll persons who use methamphetamine. We describe the protocol and baseline participant characteristics for the American Transformative HIV Study (AMETHST).

### Target Population

The AMETHST cohort study used established [59,60], web-based strategies to enroll a large sample of HIV-negative men, transmen, and transwomen who have sex with men, aged 16 to 49 years, and who are at high risk of HIV acquisition—defined as meeting objective criteria for PrEP care but *not* being on PrEP at the time of enrollment. The cohort will be followed prospectively for 36 months to examine outcomes including PrEP uptake or discontinuation, HIV acquisition, changing patterns of methamphetamine use, and rectal immune dysregulation. We aimed to enroll a cohort of participants at high risk for HIV that was geographically diverse (ie, with representation from every US state), racially and ethnically diverse (n=3031, 56.5% are participants of color), and young (n=1704, 31.8% aged 16‐29 years). We overenrolled persons who reported recent (<3 months) methamphetamine use (n=2518). All study procedures were approved by the City University of New York institutional review board, and we obtained a waiver of parental consent for those aged 16 and 17 years. Those aged 16 or 17 years were presented with additional multiple-choice questions about their comprehension of the study consent form (that they were required to answer correctly) as part of the assent process. We include those aged 16 and 17 years because data suggest that this is about the age when many men who have sex with men begin to become sexually active with other men [61], and thus vulnerable to HIV. We obtained a waiver of parental consent, given the minimal risk posed by the study and that requiring parental consent could potentially endanger participants who may not be “out” to their parents [62].

### Cohort Eligibility and Recruitment

Enrollment for AMETHST began in August 2022 and concluded in August 2023. Participants were recruited via advertisements on men-for-men geosocial sexual networking mobile phone apps. Some advertisements were more generic in nature, while others used imagery (eg, smoke exhaled from methamphetamine use) and textual cues that would suggest the study was seeking to enroll those who use methamphetamine (see Multimedia Appendix 1 for example advertisements). Of note, app developers forbid both its user base as well as advertisers from mentioning drug use directly. To subvert this, users have developed evolving coded language or emojis to communicate interest in finding meth-using partners without setting off automatic filters or censors [63,64]. Our advertisements were geotargeted to individuals using apps inside the United States. Eligibility criteria are shown in Table 1; however, once we reached saturation of individuals who do *not* use methamphetamine, we limited enrollment to just those who met these criteria and reported recent methamphetamine use (defined as at least 1 instance of use in the past 3 months). Apps themselves require users to *indicate* that they are older than 18 years of age. Individuals younger than 18 years of age are known to bypass this by saying that they are older than 18 years when they join. It is for this reason we allowed for individuals aged 16 and 17 years of age to enroll in our study (n=123).

Potential participants were directed to a secure screening survey in their device’s web browser and presented with a screen describing study participation and eliciting informed consent. The screening survey took approximately 5‐10 minutes to complete and included core eligibility criteria for the study. As the mpox outbreak was peaking in the United States at the time recruitment launched, the survey also included questions on mpox, the results of which have been published elsewhere [65]. There was no incentive for completing the screening survey. However, those who were eligible were sent a baseline survey, for which they received an incentive. Upon completion of the baseline survey, participants were mailed at-home collection kits for HIV testing (ie, oral fluid sample) and rectal swabs—all to be returned via mail to a laboratory for testing or storage. Upon receipt at the laboratory, participants received an incentive for their HIV test kit and a second incentive for returning the rectal swabs (described below).

**Table 1.** Eligibility criteria for the American Transformative HIV Study.

Eligibility criteria	Participants who used methamphetamine in the last 3 months (n=2846), n (%)	Participants who had not used methamphetamine in the last 3 months (n=2518), n (%)	Chi-square (*df*)	*P* value
Core eligibility criteria (all participants must meet these criteria)				—^a^
Aged 16 to 49 years	2846 (100)	2518 (100)	—	
At least 2 men sex partners in the past 90 days	2846 (100)	2518 (100)	—	
Not currently participating in a clinical trial for an HIV vaccine or pre-exposure prophylaxis	2846 (100)	2518 (100)	—	
Not currently on pre-exposure prophylaxis	2846 (100)	2518 (100)	—	
Never diagnosed with HIV (self-report)	2846 (100)	2518 (100)	—	
Currently residing in the United States or territories	2846 (100)	2518 (100)	—	
Not cisgender woman	2846 (100)	2518 (100)	—	
Additional eligibility criteria (participants must meet at least 1)				
≥1 receptive condomless anal sex acts with a men partner in the last 6 months	2384 (83.8)	2102 (83.5)	0.1 (1)	.78
≥3 insertive condomless anal sex acts with a men partner in the last 6 months	1913 (67.2)	1277 (50.7)	150.9 (1)	<.001
Used methamphetamine in the last 3 months	2846 (100)	0 (0)	—	—
Syphilis diagnosis in the last 6 months	149 (5.2)	55 (2.2)	34.0 (1)	<.001
Shared injection drug needles in the last 12 months	236 (8.3)	3 (0.1)	209.6 (1)	<.001

a Not applicable.

### Preventing Fraudulent Participation

We enacted multiple measures to minimize repeat participation and fraudulent participants, including recording IP addresses and using cookies to block repeated attempts. Our databases flagged duplicate contact information. Mailing addresses were validated with the US postal service, and participants were required to provide a unique valid phone number (ie, text verification). Multiple entries were identified by email addresses, mailing addresses, or phone numbers. In addition, the data manager manually checked for duplicate entries during baseline data collection. We also assessed the time to completion of our web-based surveys and checked for variability in response sets.

### Baseline Web-Based Survey

Participants were emailed and texted a unique link to a baseline survey. The web-based survey assessed psychosocial, demographic, social network, and behavioral characteristics (behaviors including sexual behavior and substance use). The full measures for the screening survey and baseline survey are included in Multimedia Appendix 2. The survey took approximately 45 minutes to complete, and participants were compensated US $40 for completing the survey.

### Biomarkers

Upon completing the baseline survey, participants were sent at-home specimen collection kits. This kit included the OraSure HIV-1 specimen collection device and 2 rectal swabs (the Aptima Multitest Swab and the Zymo DNA/RNA Shield swab). Participants were also provided access to a study video along with printed instructions on collecting their specimens. Upon receipt at the laboratory, oral fluid samples were used for HIV antibody testing using the Avioq HIV-1 enzyme immunoassay [66]. Any samples testing positive with this assay were subsequently tested with the Food and Drug Administration–cleared BioRad Geenius HIV-1/2 as a supplemental antibody test, which has been shown to reliably detect HIV-1 antibodies in oral fluid specimens with mean enzyme immunoassay signal-to-cutoff greater than or equal to 3.00 [66]. The laboratory returned all results to our team, and we in turn informed the participants. Those with HIV-negative results were informed by email, and those with HIV-reactive results were informed via phone following a published protocol [67].

Rectal swabs received by the laboratory are being banked or frozen until all follow-up assessments are complete, at which point we will be conducting a nested case-cohort study [68] to examine biological vulnerability to HIV. This is described in greater detail below. Participants were compensated US $25 for returning their HIV test kit and US $25 for returning rectal swabs.

### Participants With Unknown Baseline HIV Status

Participants who screened eligible and completed a baseline questionnaire, but did not return an HIV test kit (baseline serostatus not confirmed with a biomarker), will continue to be followed and asked at regular intervals to submit an oral fluid sample for HIV testing using the at-home sampling kit. For the purposes of prospectively estimating HIV incidence in the AMETHST cohort, these individuals will be excluded. However, we will conduct post hoc sensitivity analyses that make assumptions about having similar, lower, or higher HIV risk profiles than the AMETHST participants for whom baseline HIV status was determined. Key questions answered by including these participants will include identifying psychosocial and demographic factors associated with a timeline to sample return (for those who return a sample at a future wave) as well as how these factors differentiate those who do not return a sample going forward.

### HIV Incidence in the 12 Months Before Cohort Enrollment

We estimated pre-enrollment HIV incidence using baseline data on HIV serostatus and HIV testing history prior to joining the study. Using these data, we reconstructed a cohort of individuals who could all be classified as HIV-negative as of 12 months before cohort enrollment. For those HIV-positive at enrollment, we estimated the time of seroconversion using self-reported data on the timing of their last HIV-negative test. Specifically, those self-reporting a negative HIV test within 6 months of AMETHST enrollment were classified as being HIV-negative as of 6 months before enrollment, with seroconversion timing assumed to be distributed evenly during the 6 months leading up to study enrollment. Similarly, participants self-reporting a negative HIV test 7 to 12 months before AMETHST enrollment were classified as being HIV-negative as of 12 months before enrollment, with seroconversion timing assumed to be distributed evenly during the 12 months leading up to study enrollment. We also estimated HIV incidence for the 6-month period leading up to cohort enrollment. For the purposes of identifying boundaries around minimum and maximum incidence rates, we conducted sensitivity analyses representing assumptions for the timing of seroconversion at both extremes.

All participants are provided information on where they can access free and low-cost HIV testing outside of the study as well as information on PrEP and where they can get access to PrEP for no or low cost. Participants are also welcome to reach out to our team if they need assistance getting connected to HIV prevention resources or HIV care.

### Statistical Power

This study is sufficiently powered for all future analyses assessing the main aims of the cohort (ie, impact of methamphetamine on HIV seroconversion and PrEP uptake) considering expected HIV seroconversion rates, PrEP uptake rates, methamphetamine use, and response rates based on our prior work [69].

### Ethical Considerations

All participants provided informed consent. The AMETHST study protocol was approved by the Institutional Review Board of the City University of New York (approval 2022-0378-PHHP). Participant contact information is stored separately from their data or responses and linked only by a unique ID. At enrollment, participants were compensated US $40 for completing the survey, US $25 for returning their HIV test kit, and US $25 for returning rectal swabs.

## Results

### Overview

In total, 71,205 individuals began our screening survey, and 42,132 (59%) completed it. Of the noncompleters, most closed their browser window on the informed consent page (ie, immediately). Of the 42,132 who completed the screening survey, 9115 appeared to be eligible and were sent a baseline computer-assisted self-interview (CASI) survey, 88.3% (n=8049) of whom completed that survey. However, among those completing this baseline CASI (n=8049), 2716 were removed from further consideration (1675/2716, 61.7% of these were explicitly removed because the participant did not verify a working phone number with the study team; a measure in place to prevent fraudulent or duplicate participants).

**Figure 1.**
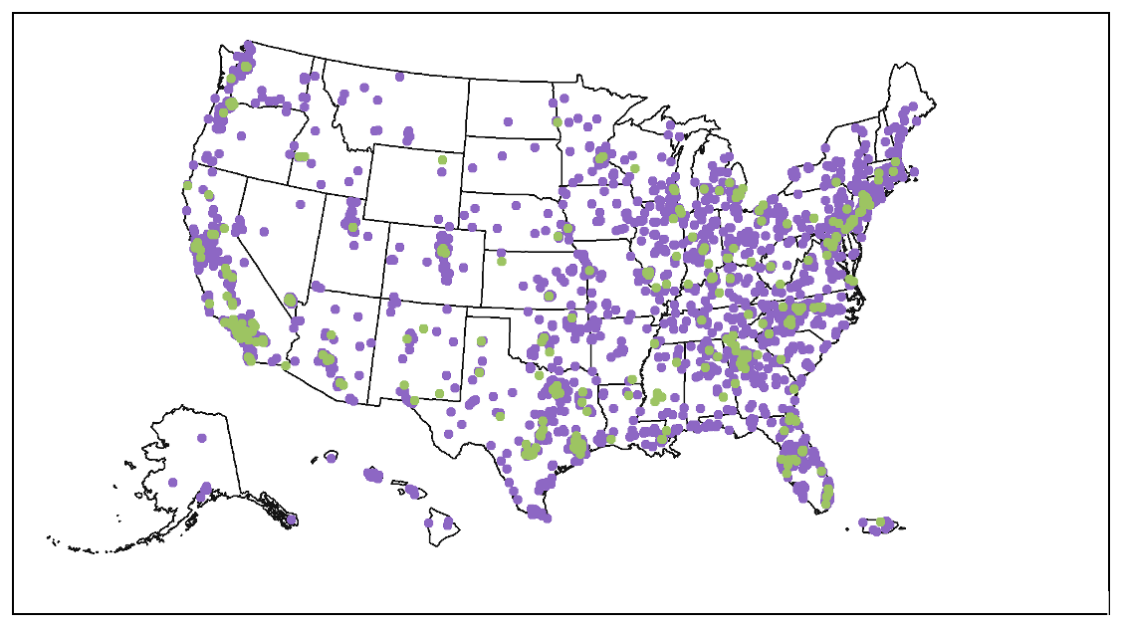
Geographic distribution of participants. Those in green had HIV-positive test results.

Of the participants who completed the baseline CASI and verified a phone number, 5364 were eligible to receive and were mailed HIV and rectal test kits. We consider these 5364 individuals to be enrolled in the cohort. Participants resided in all 50 states and Puerto Rico (Figures1  2).

Of those 5364 who were sent a kit, 78.2% returned at least 1 of the specimens (be it an HIV test kit or at least 1 rectal sample). The majority of those sent kits returned their HIV test kit (n=4054, 76%). Throughout the course of enrollment, we had to resend packages to participants a total of 1720 times (this includes 920 participants who had to have a package resent once and 800 participants who had to have a package resent more than once). Of these resent packages, 469 were partial kits (eg, an HIV test kit or a rectal swab). Reasons for resent kits varied and included participants reporting a package not having arrived to them (n=505), packages not received by the laboratory (n=71), some issue with the kit itself requiring the participant to resample (eg, insufficient sample and sample damaged in transit; n=524), the participant misplacing the kit (n=224), the participant moving or asking to send the kit to a different address (n=275), or other factors or a combination of the aforementioned (n=196). Of the participants who returned kits (n=4191), 4085 (97.5%) returned their HIV test kit and at least 1 rectal sample, 73 (1.7%) participants returned just their HIV test kits, and 33 (0.8%) participants returned at least 1 rectal sample but no HIV test kit.

As shown in Table 1, those reporting methamphetamine use were significantly more likely to report nearly all the “additional eligibility” criteria, including 3 or more insertive CAS acts with a man in the last 3 months (67.2% vs 50.7%), a syphilis diagnosis in the last 6 months (5.2% vs 2.2%), and sharing injection drug needles in the last 12 months (8.3% vs 0.1%).

**Figure 2.**
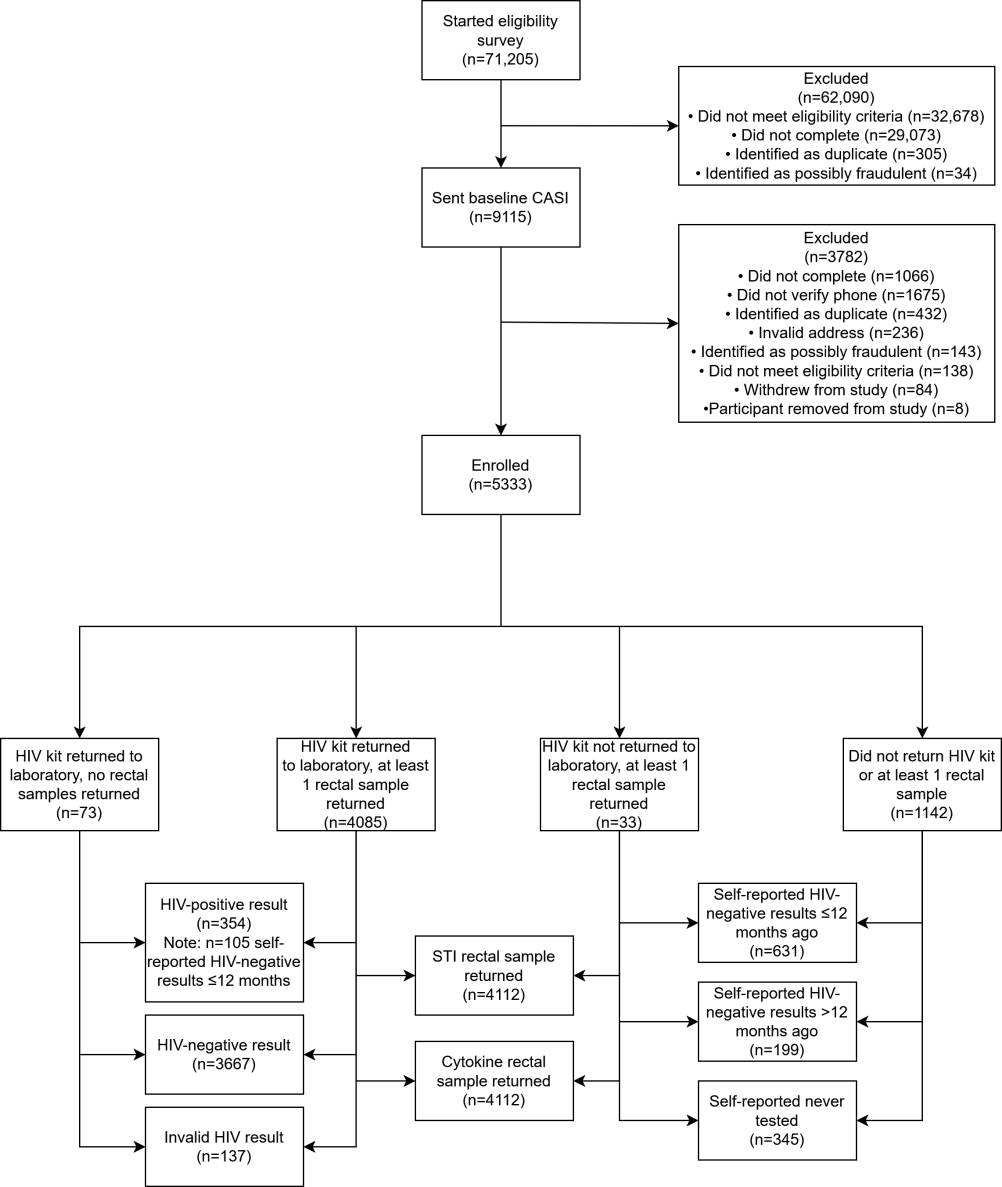
Participant flow through study procedures at enrollment. CASI: computer-assisted self-interview; STI: sexually transmitted infection.

### Baseline Characteristics of the AMETHST Cohort

Nearly all (4542/5364, 84.7%) were cisgender men, 3.7% (201/5364) were transgender women, and 1.1% (61/5364) were transgender men who have sex with men (Table 2). There were also 560 (10.4%) individuals who self-identified outside of the gender binary—all reported being assigned male sex at birth. In total, 56.5% (3031/5364) were persons of color, and 31.8% (1714/5364) were younger individuals aged 16 to 29 years.

As shown in Table 2, those reporting methamphetamine use were more likely to be White, transgender women or nonbinary, nongay identified, older, and to have never heard of PrEP. They were less likely to have health insurance, have a primary care provider, and were significantly more likely to have tested for HIV more than a year ago or had never been tested for HIV in their lives.

**Table 2.** Demographic characteristics for the American Transformative HIV Study cohort study.

	Participants who used methamphetamine in the last 3 months (n=2846), n (%)	Participants who had not used methamphetamine in the last 3 months (n=2518), n (%)	Chi-square (*df*)	*P* value
Race or ethnicity			242.8 (4)	<.001
Asian or Pacific Islander	37 (1.3)	153 (6.1)		
Hispanic or Latinx	649 (22.8)	829 (32.9)		
Non-Hispanic Black	350 (12.3)	416 (16.5)		
Non-Hispanic White	1452 (51)	881 (35)		
Multiracial or other	358 (12.6)	239 (9.5)		
Gender			40.5 (3)	<.001
Cisgender male	2342 (82.3)	2200 (87.4)		
Transgender woman	137 (4.8)	64 (2.5)		
Transgender man	25 (0.9)	36 (1.4)		
Nonbinary or other	342 (12)	218 (8.7)		
Sexual identity			123.8 (2)	<.001
Gay	1760 (61.9)	1912 (75.9)		
Bisexual	664 (23.3)	355 (14.1)		
Other	422 (14.8)	251 (10)		
Age (years)			240.3 (6)	<.001
16‐19	31 (1.1)	92 (3.7)		
20‐24	179 (6.3)	369 (14.7)		
25‐29	467 (16.4)	566 (22.5)		
30‐34	731 (25.7)	613 (24.3)		
35‐39	630 (22.1)	438 (17.4)		
40‐44	505 (17.7)	273 (10.8)		
45‐49	303 (10.6)	167 (6.6)		
Has health insurance	1947 (68.4)	1989 (79)	76.5 (1)	<.001
Has a primary care provider	1174 (41.3)	1505 (59.8)	183.3 (1)	<.001
Never heard of PrEP^a^	83 (2.9)	41 (1.6)	9.8 (1)	.001
HIV testing history			175.9 (3)	<.001
In the last 6 months	882 (31)	1017 (40.4)		
In the last 7‐12 months	862 (30.3)	940 (37.3)		
More than a year ago	882 (31)	417 (16.6)		
Never been tested for HIV	220 (7.7)	144 (5.7)		

a PrEP: pre-exposure prophylaxis.

### Completion of Baseline HIV Testing and Returning Rectal Swabs

All 5364 participants enrolled in the cohort were mailed specimen collection kits (oral fluid for HIV testing and 2 rectal swabs) via United States Postal Service, of which 74.5% (3998/5364) returned both their HIV test kit and at least 1 rectal sample. An additional 3.6% (194/5364) partially returned their samples (either an HIV test kit or at least 1 rectal sample). The remaining did not return their kits, and failure to return kits was higher among those who reported methamphetamine use (28.6% vs 14.3%) despite being sent multiple reminders to do so. Among those having returned an HIV test kit, 3.3% were HIV-positive among those who did not report methamphetamine use, and 15.5% were HIV-positive among those reporting methamphetamine use. Among those with HIV-negative results (n=3683), those reporting methamphetamine use were significantly more likely to report their last HIV test was either more than a year ago (27% vs 15.5%) or having either never tested for HIV or not knowing the results of their last test (6.5% vs 3.9%). In contrast, among those with HIV-positive results (n=371), there were no significant differences in how long ago they previously tested HIV-negative (Table 3).

**Table 3.** Biomarkers and HIV results for the American Transformative HIV Study.

	Participants who used methamphetamine in the last 3 months (n=2846), n (%)	Participants who had not used methamphetamine in the last 3 months (n=2518), n (%)	Chi-square (*df*)	*P* value
Returned sampling kits to the laboratory			185.2 (2)	<.001
Did not return either kit	813 (28.6)	359 (14.3)		
Partial kit return (HIV or rectal)	128 (4.5)	66 (2.6)		
Returned all kits (HIV and at least 1 rectal)	1905 (66.9)	2093 (83.1)		
HIV test kit result (only those with valid HIV test kit results, n=4054)			183.1 (1)	<.001
HIV-negative	1642 (84.5)	2041 (96.7)		
HIV-positive	302 (15.5)	69 (3.3)		
Date of most recent HIV test (only those with valid HIV-negative results, n=3683)			95.4 (3)	<.001
Self-reported HIV-negative test <6 months ago	585 (35.6)	843 (41.3)		
Self-reported HIV-negative test 7‐12 months ago	507 (30.9)	801 (39.2)		
Self-reported HIV-negative test >12 months ago	443 (27)	317 (15.5)		
No previous HIV test, indeterminate result, or do not know or do not remember	107 (6.5)	80 (3.9)		
Date of most recent HIV test (only those with valid HIV-positive results, n=371)			4.7 (3)	.19
Self-reported HIV-negative test<6 months ago	54 (17.9)	13 (18.8)		
Self-reported HIV-negative test 7‐12 months ago	86 (28.5)	23 (33.3)		
Self-reported HIV-negative test >12 months ago	137 (45.4)	23 (33.3)		
No previous HIV test, indeterminate result, or do not know or do not remember	25 (8.3)	10 (14.5)		

### HIV Status and Estimating the Cohort HIV Incidence Rate Before AMETHST Enrollment

Based on participants’ HIV results as well as self-reporting when their most recent HIV-negative test was, we estimated that the incidence rate in this cohort in the 12-month period leading up to study enrollment was 10.06 (95% CI 8.65‐11.64) per 100 person-years among those reporting methamphetamine use compared with 2.04 (95% CI 1.49‐2.73) among those not reporting methamphetamine use per 100 person-years. The estimate for the 6-month period leading up to enrollment was 8.85 (95% CI 7.02‐11.01) per 100 person-years among those reporting methamphetamine use compared with 1.85 (95% CI 1.14‐2.83) among those not reporting methamphetamine use per 100 person-years (Table 4).

**Table 4.** HIV incidence among American Transformative HIV Study participants in the 12- and 6-month periods before study enrollment.

	Participants who used methamphetamine in the last 3 months (n=2820)	Participants who had not used methamphetamine in the last 3 months (n=2584)	Incidence rate ratio
12-month period before enrollment			
Presumed recent seroconversions, n^a^	174	42	—^b^
Person-years at risk among people who seroconverted^c^, n	87	21	—
Person-years at risk among persons with HIV-negative test results, n	1642	2041	—
Incidence rate per 100 person-years (95% CI)^d^	10.06 (8.65-11.64)	2.04 (1.49-2.73)	4.93 (3.55-6.99)
6-month period before enrollment			
Presumed recent seroconversions, n^e^	76	19	—
Person-years at risk among people who seroconverted^c^, n	38	9.5	—
Person-years at risk among persons with HIV-negative test results, n	821	1020.5	—
Incidence rate per 100 person-years (95% CI)^d^	8.85 (7.02-11.01)	1.85 (1.14-2.83)	4.78 (2.94-8.12)

a To accurately approximate the number of recent seroconversions for the 12-month incidence calculation, we first totaled the number of HIV-positive results for testing categories ≤6 months and 7‐12 months (Table 3). Then, we added 25% of the adjacent category of those who reported last testing >12 months ago.

b Not applicable.

c Person-years calculated as (1) mid-year estimates for those presumed to have seroconverted in the last year (12-month incidence rate), (2) full-year estimates for people who did not seroconvert (12-month incidence rate), (3) quarter-year estimates for those presumed to have seroconverted in the last 6 months (6-month incidence rate), and (4) half year estimates for those who did not seroconvert (6-month incidence rate).

d Mid-P exact test 95% CIs calculated using OpenEpi software.

e To accurately approximate the number of recent seroconversions for the 6-month incidence calculation, we used the number of HIV-positive results for the ≤6 months category. Then, we added 25% of those who reported the last testing 7-12 months ago.

### Longitudinal Follow-Up and Measurement

Prospective closed follow-up of AMETHST participants includes completion of web-based surveys beginning 12 months after the baseline survey, again at 24 months, and finally at 36 months, as well as annual self-administered at-home HIV test and rectal swabs. However, at follow-up, participants who self-report being on PrEP (ie, PrEP adoption outside of the study) are asked to provide proof in the form of a photo of the medication bottle with their prescription information or other medical documentation of their prescription. Likewise, those who report having been diagnosed with HIV between study assessments are asked to provide proof in the form of a picture of a document or medication bottle with their prescription information. Those known to be HIV-positive (either via testing HIV-positive during a prior wave of assessments or documented proof of HIV seroconversion between study assessments) will not be asked to test for HIV within the study again. Our reason for not asking those on PrEP to test with us is because our test is a third-generation HIV test, and those on PrEP should be receiving a fourth-generation HIV test (blood) from their medical provider, and we do not want participants to inadvertently use our test in lieu of their providers. Should participants report discontinuing PrEP at a follow-up assessment, they will be asked to test with us again.

A comprehensive list of key study measurements by study wave is included in Multimedia Appendix 3. We will follow the cohort of 5364 individuals to characterize methamphetamine use or uptake or discontinuation and its role in the following: the rate of PrEP uptake or discontinuation, individual or network or contextual-level determinants of PrEP uptake or discontinuation, patterns of PrEP use (eg, daily or on-demand), the rate of new HIV seroconversions and other missed HIV prevention opportunities (ie, sexually transmitted infections while not on PrEP), individual or network or contextual-level determinants of HIV seroconversion and missed HIV prevention opportunities, racial or ethnic disparities in HIV incidence and their trends over time, and the influence of PrEP uptake on racial or ethnic disparities in HIV incidence.

Given the nature of this web-based US nationwide cohort, we elected to longitudinally follow all participants who mailed an HIV test kit at baseline rather than only those who completed HIV testing. Participants who did not complete HIV testing will have the opportunity to provide those data or samples at future assessments, and this will enable us to learn more about differential rates of participation and attrition in the cohort moving forward.

### Biological Vulnerability to HIV—Nested Case-Cohort Study

At the completion of all follow-up assessments, we will conduct a nested case-cohort study [68] to examine biological vulnerability to HIV. We anticipate that upward of 450 individuals will have seroconverted to HIV-positive, and we will match their samples against 450 HIV-negative participants (similar in age and race). The rectal swabs for these 900 participants will be retrieved from the biospecimen repository. For those who are HIV seroconvert, swabs preceding seroconversion will be selected. One swab (the Aptima Multitest Swab) will be tested for rectal gonorrhea and chlamydia. The second swab (the Zymo DNA/RNA Shield swab) will be examined using the LEGENDPlex assays to quantify rectal cytokine or chemokine levels. We will use the Human Inflammation Panel, which allows for the measurement of 13 cytokines or chemokines [70]. This will allow to determine changes in cytokine or chemokine profiles before and after HIV seroconversion—matched against those of HIV-negative participants—and to determine if methamphetamine use is associated with any immune changes in the rectal mucosa among those who acquire HIV as well as in the comparison group.

## Discussion

### Principal Findings

The AMETHST cohort study enrolled an entirely web-based US national cohort of cisgender men, transgender men, and transgender men who have sex with men and are at very high risk for HIV, into longitudinal follow-up. Greater than half of the participants were persons of color, and 53.1% had recently used methamphetamine. Importantly, none of the AMETHST participants were on PrEP at enrollment, but all met the clinical criteria for PrEP use. This will allow our study to fill critical knowledge gaps that can improve our understanding of the role that methamphetamine plays in (barriers to) PrEP uptake or discontinuation among those most in need of HIV prevention interventions—and at a time in which PrEP has been Food and Drug Administration–approved for more than a decade.

Although all participants were similar in their having met clinical criteria for PrEP, we observed several characteristics that distinguished people who used methamphetamine at baseline from those who did not in ways that put them at greater risk for HIV. People who used methamphetamine were more likely to report greater than 3 recent insertive CAS acts with men, to have been diagnosed recently with syphilis, to report sharing injection drug needles in the past year, trended toward being older, having never tested for HIV, and to not have health insurance, nor a primary care provider, or have heard of PrEP. These facets all compound to elevate HIV risk. This is perhaps evidenced best through the staggering number of HIV infections (baseline prevalence) identified among those who use methamphetamine (n=302, 15.5% vs n=69, 3.3% among those not using). This high number was despite lower HIV kit return among those who use methamphetamine. Our baseline data highlight the challenges people who use methamphetamine face as well as the challenges we as researchers may have in engaging and retaining this population longitudinally. Our estimates of HIV incidence in the 12-month period leading up to enrollment (2.04% per year among those who do not use methamphetamine and 10.06% per year among those who use methamphetamine) suggest that, without significant PrEP uptake as the cohort moves forward, the risk of HIV acquisition that we will observe prospectively will also be quite high. As noted, per enrollment criteria, no participants were taking PrEP at the time of enrollment; however, we provide participants information and resources on how to access PrEP and will be monitoring that outcome going forward.

### Strengths

Major strengths of the AMETHST cohort include its large sample size, geographic diversity, self-reported data related to substance use, HIV risk and PrEP, biomarkers for direct measurement of HIV status and seroconversion and inflammation, recruitment of participants independent of their access to or engagement with the health care system, and inclusion of large numbers of racial or ethnic minority SGM individuals as well as those younger than the age of 29 years. Having overrepresented persons who use methamphetamine uniquely positions this cohort to answer key causal questions about the role that methamphetamine plays in disrupting HIV prevention. As well, our future nested case-cohort study will enable our team to answer key questions regarding the biological role that methamphetamine plays in HIV seroconversion.

Importantly, the AMETHST cohort study design, with its infrequent (ie, annual) surveys, reduces the potential for participation and questionnaire-response bias introduced by the Hawthorne effect. Studies involving frequent contact can cause participants to adopt behaviors that make them less representative of the vulnerable populations from which they were drawn. For example, McCambridge et al [71] discussed how research participant effects—an expansion on the Hawthorne effect [72]—can introduce bias, including demand characteristics [73]. Studies involving high levels of staff contact with participants may induce behavior change by repeatedly engaging participants outside of their natural context, artificially impacting results and reducing generalizability [74,75]. That being said, we recognize that any self-reporting of a behavior introduces some potential for a participant to reflect upon, and thus modify, their behavior.

### Limitations

Recruiting participants and gathering data on the web are a less controlled research environment than face-to-face studies. Web-based recruitment increases vulnerability to repeat participation and fraudulent manipulation of HIV testing procedures. We took several measures to avoid this, including requiring all participants to have a unique email address, a unique and valid phone number (with text verification), and a valid mailing address (verified with the US postal service). We tracked IP addresses and blocked responses from outside the United States on our screening survey. Our advertising materials did not indicate full eligibility criteria (eg, age 16 to 49 years, cannot be HIV-positive, and meeting criteria for PrEP care but not being on PrEP), and the screening survey included additional items not related to study eligibility such as cannabis use, mpox infection or vaccination, health insurance status, and doxycycline postexposure prophylaxis. However, no method is entirely infallible. At future waves of follow-up, data will be compared to responses at baseline for consistency (eg, age and race).

Next, participants were recruited via sexual networking apps and are thus not representative of all SGM individuals highly vulnerable to HIV. These apps have emerged as the modal means through which SGM individuals meet sex partners and have become an essential tool for researchers and providers to reach this population.

Finally, HIV seroconversions and PrEP uptake may be incompletely ascertained, and the timing of HIV seroconversions must be estimated using a midpoint approach with broad intervals, potentially introducing bias in our baseline HIV incidence estimates. For example, our study eligibility or inclusion criterion of never diagnosed with HIV (self-report) effectively excludes frequent HIV testers who seroconverted and were diagnosed in the period immediately before the study launch.

### Conclusions

The AMETHST cohort is a large racially and geographically diverse group of SGM individuals who report high vulnerability to HIV. Its focus to identify multilevel (ie, structural, psychological, and social) and biobehavioral (ie, rectal cytokines or chemokines) determinants of amplified HIV seroconversion risk for SGM individuals, including those who use methamphetamine, will serve to guide targeted, combination interventions to reduce HIV incidence and increase PrEP uptake. Its added nested case-cohort study [68] will determine changes in cytokine or chemokine profiles before and after HIV seroconversion as well as the role that methamphetamine plays in that process. Our team welcomes new collaborations both in secondary analysis of the data generated as well as proposals for new measures or data to be gathered from participants in future waves of follow-up. Instructions and a concept proposal form are available by emailing the principal investigators (CG and AWC). Submitted concept proposals will be reviewed by the principal investigators (CG and AWC) and a core group of AMETHST investigators with rapid turnaround.

## Supplementary material

10.2196/66921 Multimedia Appendix 1 Sample recruitment advertisements.

10.2196/66921 Multimedia Appendix 2 Baseline survey.

10.2196/66921 Multimedia Appendix 3 Screening survey.

## Abbreviations

AMETHST: American Transformative HIV Study
CAS: condomless anal sex
CASI: computer-assisted self-interview
PrEP: pre-exposure prophylaxis
SGM: sexual and gender minority
