# Supplementary material for: The American Transformative HIV Study: Protocol for a US National Cohort of Sexual and Gender Minority Individuals With HIV
Source: JMIR Public Health Surveill. 2025 May 22;11:e66921. doi: 10.2196/66921 (PMC12121540; doi:10.2196/66921)
Supplement: Multimedia Appendix 1 [file publichealth-v11-e66921-s001.docx]

Multimedia Appendix – Sample Ads


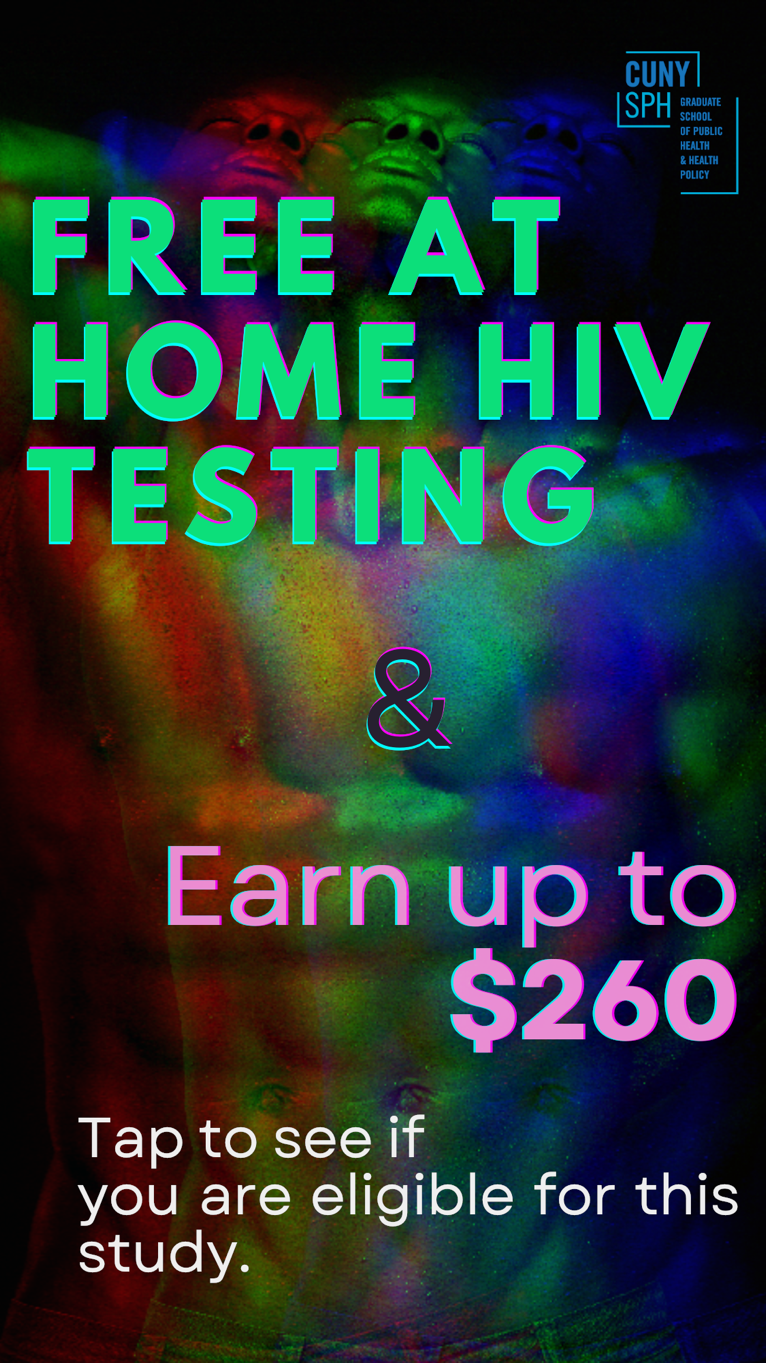


Figure 1: Generic recruitment advertisement


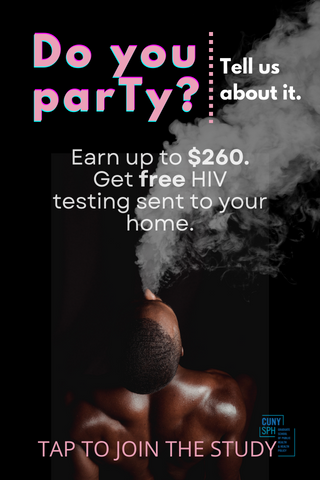


Figure 2: Meth-tailored recruitment advertisement
