# Supplementary material for: The American Transformative HIV Study: Protocol for a US National Cohort of Sexual and Gender Minority Individuals With HIV
Source: JMIR Public Health Surveill. 2025 May 22;11:e66921. doi: 10.2196/66921 (PMC12121540; doi:10.2196/66921)
Supplement: Multimedia Appendix 2 [file publichealth-v11-e66921-s002.docx]

AMETHST Baseline Survey

Contents

[Demographics 3](#_Toc110241626)

[HIV and STI Diagnosis and Treatment History 6](#_Toc110241627)

[Sexual Behavior with Main and Casual Partners 7](#_Toc110241628)

[Perceived Risk of HIV Scale 9](#_Toc110241629)

[PrEP 11](#_Toc110241630)

[2-1-1 Dosing 15](#_Toc110241631)

[Antibiotic and Dietary Supplement Use 17](#_Toc110241632)

[Food Insecurity 19](#_Toc110241633)

[ASSIST 20](#_Toc110241634)

[ASSIST 1 20](#_Toc110241635)

[ASSIST 2 21](#_Toc110241636)

[ASSIST 3 24](#_Toc110241637)

[ASSIST 4 25](#_Toc110241638)

[ASSIST 5 26](#_Toc110241639)

[ASSIST 6 27](#_Toc110241640)

[ASSIST 7 28](#_Toc110241641)

[ASSIST 8 29](#_Toc110241642)

[PNP 30](#_Toc110241643)

[Substance Use Networks 31](#_Toc110241644)

[AUDIT-10 Alcohol 33](#_Toc110241645)

[Self Perceived Need for Treatment 35](#_Toc110241646)

[CES-D 10 Depression 36](#_Toc110241647)

[GAD-7 Anxiety 37](#_Toc110241648)

[Hypersexual Disorder Inventory 38](#_Toc110241649)

[Main and Other Partners 39](#_Toc110241650)

[Felt Stigma 41](#_Toc110241651)

[Internalized Homophobia 43](#_Toc110241652)

[Sexual Minority Stress 44](#_Toc110241653)

[Substance Use Stigma 45](#_Toc110241654)

[Injection Drug Use 46](#_Toc110241655)

[Insurance 48](#_Toc110241656)

[PTSD-5 51](#_Toc110241657)

[Everyday Experiences of Discrimination 52](#_Toc110241658)

[Brief Connor-Davidson Resilience Scale (CD-RISC-10) 53](#_Toc110241659)

[Multidimensional Scale of Perceived Social Support 54](#_Toc110241660)

[Childhood Sexual Abuse 55](#_Toc110241661)

[Intimate Partner Violence 56](#_Toc110241662)

[Substance Use Treatment 57](#_Toc110241663)

[Acculturation 61](#_Toc110241664)

[Douching 63](#_Toc110241665)

[Contact Information 65](#_Toc110241666)

## Demographics

**BirthDate.** What is your date of birth? (Please use MM/DD/YYYY, e.g. 11/29/1987)

1. [full birth date entry]

**Employ**. Which of the following best describes your current employment status?

1. Full-time (40 hours per week)
2. Part-time (less than 40 hours per week)
3. Part-time work - full time student
4. Permanent or temporary disabled and **not** working
5. Permanent or temporary disabled **but** working “off the books” (or "under the table")
6. Unemployed -- Student
7. Unemployed – Other

**Income**. Which best describes your total yearly income during the last year?

1. Less than $10,000
2. $10,000 to $19,999
3. $20,000 to $29,999
4. $30,000 to $39,999
5. $40,000 to $49,999
6. $50,000 to $74,999
7. $75,000 to $99,999
8. $100,000 to $149,999
9. $150,000 to $199,999
10. $200,000 to $249,999
11. $250,000 or more

**Educ**. What's the highest level of education that you have completed?

1. 8^th^ Grade or less
2. 9^th^ Grade
3. 10^th^ Grade
4. 11^th^ Grade
5. High School Diploma or GED
6. Some College or Associates Degree
7. Currently enrolled in college
8. 4-Year College Degree (BA, BS, BFA)
9. Some Graduate School
10. Master's Degree
11. Doctorate Degree

**Height.** What is your height in feet and inches?

1. ______ feet **[VALIDATED < 8]**
2. ______ inches **[VALIDATED < 12]**

**Weight.** What is your weight (in lbs)?

1. [Text entry validated as a number between 50 and 700 lbs]

**Housing**. What is your current living situation? **Please select all that apply**.

1. Alone [EXCLUSIVE]
2. With Roommates
3. With Family
4. With Partner (spouse, boyfriend, girlfriend)
5. Other (Please specify):

**HousingInstYear**. In the **last year**, has there been a period of time in your life where you were unstably housed (e.g., couch-surfing, homeless)? **Please select all that apply.**

- 1. No [EXCLUSIVE]
  2. Yes, in a car
  3. Yes, couch-surfing
  4. Yes, in a shelter
  5. Yes, homeless
  6. Yes, other (please specify):

**SexWorkYear**. Have you exchanged sex for money, drugs, or a place to stay in the **last year**?

1. Yes, I’ve been paid for sex
2. Yes, I have paid for sex
3. Yes, I’ve **both** been paid **and** have paid for sex
4. No

**Incarcerated.** Have you ever been incarcerated (prison, jail, or juvenile detention)?

1. Yes
2. No

**YearOut**. In what year were you released from incarceration?

1. (Drop down year list)

**Arrested**. Have you been arrested in the **past 3 months**?

1. Yes
2. No

**BirthCountry.** Where were you born?

1. In the United States (i.e. one of the 50 states)
2. In a United States territory (e.g.. Puerto Rico, Guam, American Samoa, Northern Mariana Islands, U.S. Virgin Islands, etc.).
3. Outside the U.S. or its territories

**[IF BirthCountry = In the US DISPLAY BirthState]**

**BirthState.** Which US state were you born in?

1. [Drop down of 50 US states]

**[IF BirthCountry = US territory DISPLAY BirthTerritory]**

**BirthTerritory.** Which US territory were you born in?

1. [Drop down of US territories]

**[IF BirthCountry = Outside the US DISPLAY BirthOutside AND WhenCame]**

**BirthOutside**. Where were you born outside of the United States?

1. [Drop down of non-US countries]

**[IF BirthOutside=Other DISPLAY BirthOutside_Other]**

**BirthOutside_Other** Please specify what country you were born in.

1. [ Text Entry]

**WhenCame.** If you were not born in the United States, how old were you when you first came to live here full-time?

1. [validated number entry]

## HIV and STI Diagnosis and Treatment History

**HIVStatus**. What is your HIV status?

1. HIV-negative
2. HIV-positive
3. I don’t know; I am unsure

**[DISPLAY HIVdxDate if HIVStatus is HIV POSITIVE]**

**HIVdxDate**. When were you diagnosed with HIV?

1. Year
2. Month

**[DISPLAY PastHIVSimple to TestFreq if HIVStatus is Negative or Don’t Know]**

**PastHIVSimple**. When was the **last** time you were tested for HIV?

1. Less than 6 months ago
2. 6-12 Months Ago
3. More than 12 months ago
4. Never (I have never been tested for HIV)

**LastHIVOutNew**. Where did you get this testing done?

1. A doctor’s office, medical provider (non-emergency services)
2. Hospital, urgent care, or emergency services
3. A community based organization / department of health / STD clinic
4. Health Fair / Testing Van / Social Event
5. As a part of this study
6. As part of another research study
7. An at-home testing kit
8. Other (Please specify):

**TestFreq**. How often do you typically get tested for HIV?

1. Less than once a year
2. Once a year
3. Twice a year
4. Three times a year
5. Four times a year
6. Five times a year
7. Six times a year
8. Seven or more times a year

## Sexual Behavior with Main and Casual Partners

**FirstOralMale**. How old were you when you first had consensual oral sex with a male?

1. Enter age [TEXT BOX] **[validated to less than or equal to current age]**
2. I have never done this

**FirstAnalMale**. How old were you when you first had consensual anal sex with a male?

1. Text entry validated to at or below current age

**FirstOralFemale**. How old were you when you first had consensual oral sex with a female?

1. Text entry **validated to at or below current age**
2. I have never done this

**FirstAnalFemale**. How old were you when you first had consensual anal sex with a female?

1. Text entry **validated to at or below current age**
2. I have never done this

**SexPos**. Which sexual position do you identify most as?

- 1. Top
  2. Versatile/Top
  3. Versatile
  4. Versatile/Bottom
  5. Bottom
  6. Not applicable to me

**Cis (cisgender) refers to someone who currently identifies with the sex they were assigned at birth.**

**Trans (transgender) refers to someone who currently identifies differently than the sex they were assigned at birth**

**MalePartners3m**. In the **past 3 months**, how many **cis men** (**i.e., not trans men)** have you had anal sex with?

1. [number entry]

**FemalePartners3m**. In the **past 3 months**, how many **cis women (i.e. not trans women**) have you had **anal/vaginal** sex with?

1. [number entry]

**PastRecep** When was the most recent time you had **receptive** anal sex, i.e. bottomed (with or without a condom)?

1. Never
2. In the last day
3. 1-3 days ago
4. 4-10 days ago
5. 11-30 days ago
6. More than 30 days ago.

**PastURecep**. When was the most recent time someone ejaculated (came) inside your butt?

1. Never
2. In the last day
3. 1-3 days ago
4. 4-10 days ago
5. 11-30 days ago
6. More than 30 days ago.

**[DISPLAY UAVIFemale IF FemalePartners > 0]**

**UAVIFemale3m**. How many times have you had anal/vaginal sex without a condom with a female partner in the **past 3 months**?

1. [number entry]

**TransmenPartners3m**. In the **past 3 months**, how many trans men have you had anal/vaginal sex with?

1. [number entry]

**TranswomenPartners3m**. In the **past 3 months**, how many trans women have you had anal/vaginal sex with?

1. [number entry]

**URAI3m**. In the **past 3 months**, how many times did you have **receptive anal sex** (you were the bottom) with a **cis man (i.e., not a trans man)** **without a condom?**

1. [number entry]

**PozMalePartners3m**. In the **past 3 months**, how many of your male sex partners were **HIV-positive?**

1. [number entry]

**[DISPLAY URAIPos if URAI and PozMalePartners ARE NOT 0]**

**URAIPos3m.** In the **past 3 months**, how many times did you have receptive anal sex (you were the bottom) **without a condom with a cis man (i.e., not a trans man)** who was **HIV-positive?**

1. [number entry]

**UIAI3m.** In the **past 3 months**, how many times did you have **insertive anal sex** (you were the top) **without a condom** with a **cis man (i.e., not a trans man)?**

1. [number entry]

**[DISPLAY UIAIPos if UIAI and PozMalePartners ARE NOT 0]**

**UIAIPos3m**. In the **past 3 months**, how many times did you have **insertive anal sex** (you were the top) **without a condom** with a **cis man (i.e., not a trans man)** who was **HIV-positive?**

1. [number entry]

## Perceived Risk of HIV Scale

[**DISPLAYED TO ALL HIV NEGATIVE PARTICIPANTS]**

**PRHS1**. What is your gut feeling about how likely you are to get infected with HIV?

1. Extremely unlikely
2. Very unlikely
3. Somewhat likely
4. Very likely
5. Extremely likely

**PRHS2**. I worry about getting infected with HIV.

1. None of the time
2. Rarely
3. Some of the time
4. A moderate amount of time
5. A lot of the time
6. All of the time

**PRHS3**. Picturing self getting HIV is something I find:

1. Very hard to do
2. Hard to do
3. Easy to do
4. Very easy to do

**PRHS4**. I am sure I will **NOT** get infected with HIV.

1. Strongly disagree
2. Disagree
3. Somewhat disagree
4. Somewhat agree
5. Agree
6. Strongly agree

**PRHS5**. I feel vulnerable to HIV infection

1. Strongly disagree
2. Disagree
3. Somewhat disagree
4. Somewhat agree
5. Agree
6. Strongly agree

**PRHS6**. There is a chance, no matter how small, I could get HIV

1. Strongly disagree
2. Disagree
3. Somewhat disagree
4. Somewhat agree
5. Agree
6. Strongly agree

**PRHS7**. I think my chances of getting infected with HIV are:

1. Zero
2. Alost zero
3. Small
4. Moderate
5. Large
6. Very large

**PRHS8.** Getting HIV is something I have…

1. Never thought about
2. Rarely thought about
3. Thought about some of the time
4. Thought about often

## PrEP

[**DISPLAY PrEP BLOCK FOR ALL HIV NEGATIVE PARTICIPANTS]**

PrEP (pre-exposure prophylaxis) is a new biochemical strategy to prevent HIV infection. PrEP involves HIV-negative people taking anti-HIV medications (for example, Truvada) once a day, every day to reduce the likelihood of HIV infection if they were exposed to the virus. PrEP is highly effective to reduce the likelihood of HIV infection.

Please note that PrEP is not the same as taking HIV medications for a brief period of time (i.e., 28 days) after a high risk exposure to HIV through encounters such as being stuck by a contaminated needle or having unprotected intercourse. PrEP is intended for regular, long-term use.

**[IF PrEPstatus CURRENTLY ON PrEP send emailtrigger]**

**PrEPstatus**. Have you ever taken PrEP (Pre-Exposure Prophylaxis) to prevent HIV (e.g. Truvada, Descovy, Apretude, or generic PrEP)?

1. Yes, I am currently on PrEP
2. Yes, but I am not currently taking PrEP
3. No, never taken PrEP
4. I don’t know what PrEP is

**[If PrEPstatus is CURRENTLY ON PrEP display PrEPWhen]**

**PrEPWhen** When did you most recently begin PrEP?

1. Within the last week
2. 1-2 weeks ago
3. 3-4 weeks ago
4. 1-2 months ago
5. 3-6 months ago
6. 7-12 months ago
7. More than a year ago

**HearPrEP**. Where did you first hear about PrEP?

1. As part of this study
2. As part of my participation in a different research study
3. Through a news media source
4. Through a social media source
5. Through a friend
6. Through my main partner
7. Through a casual sex partner
8. Through a family member
9. Through a medical provider
10. Through a community-based agency
11. Other (Please describe): _______________________
12. I don’t remember

**PrEPview**. What source has had the biggest influence on your current views about PrEP?

1. As part of this study
2. As part of my participation in a different research study
3. Through a news media source
4. Through a social media source
5. Through a friend
6. Through my main partner
7. Through a casual sex partner
8. Through a family member
9. Through a medical provider
10. Through a community-based agency
11. Other (Please describe): ________________________
12. I don’t remember

**[If PrEPstatus is NOT Currently On PrEP display SpokeDocPrEP]**

**SpokDocPrEP**. Have you ever spoken to a medical provider about starting PrEP?

1. Yes, and we both decided it was a good option for me and I should start PrEP
2. Yes, and we both decided it might be a good option but to wait before beginning PrEP
3. Yes, and we both decided it was not a good option for me
4. Yes, and the provider was not comfortable prescribing PrEP for me
5. Yes, and the provider thought it was a good option but I chose not to do it
6. No, I have not ever spoken to a provider about starting PrEP

**[DISPLAY PrEPNotSpok IF SpokDocPrEP = No]**

**PrEPNotSpok**. What are the reasons you have not yet spoken to a medical provider about PrEP? Check all that apply.

- 1. I did not know what PrEP was
  2. I am unsure if I am at high enough risk for HIV
  3. I don’t have a way to pay for it
  4. I haven’t don’t have access to a medical provider who will prescribe it
  5. I haven’t seen my medical provider recently
  6. I haven’t told my medical provider that I am gay/bi/trans
  7. I am concerned about talking to my medical provider about my sex life
  8. I am concerned about PrEP side effects
  9. I prefer to use condoms.
  10. Other (Please specify):_____________________________________________

**PrEPCand**. Do you believe that you are currently an appropriate candidate for PrEP?

1. Yes, I am definitely an appropriate candidate for PrEP
2. Yes, I think I am an appropriate candidate for PrEP
3. I’m not sure if I am an appropriate candidate for PrEP
4. No, I don’t think I am an appropriate candidate for PrEP
5. No, I am definitely not an appropriate candidate for PrEP

**PrEPFav**. In general, I would say the people I am close with:

1. Are in favor of PrEP
2. Are opposed to PrEP
3. Are split evenly between being in favor of and being opposed to PrEP
4. Don’t have strong opinions either way
5. Don’t really know what PrEP is

**[Display PrEPLike to PrEPinject if PrEPstatus is NOT Currently On PrEP]**

**PrEPLike**. Research shows that PrEP is at least 99% effective in preventing HIV through sex when taken daily. How likely would you be to take PrEP if it were available for free?

1. I would definitely take it
2. I would probably take it
3. I might take it
4. I would probably not take it
5. I would definitely not take it

**PrEPPlan**. PrEP is currently available with a prescription from your doctor, and research has shown that a majority of insurance companies cover most or all of the costs of PrEP. Do you plan to begin PrEP?

1. Yes, I will definitely begin taking PrEP
2. Yes, I will probably begin taking PrEP
3. I’m not sure – I might begin taking PrEP
4. No, I probably will not begin taking PrEP
5. No, I definitely will not begin taking PrEP

**[DISPLAY PrEPSoon IF PrEPPlan = A or B]**

**PrEPSoon**. How soon do you plan to begin taking PrEP?

- 1. Within the next month
  2. Within the next 2-3 months
  3. Within the next 4-6 months
  4. Within the next 7 months to a year
  5. More than a year from now

**[DISPLAY PrEPReas if PrEPPlan = C, D, or E]**

**PrEPReas**. What are the reasons you do not intend to begin PrEP? Check all that apply:

- 1. I’m not at high enough risk for HIV
  2. I don’t have a way to pay for it
  3. I don’t have access to a medical provider who will prescribe it
  4. I don’t believe it works well enough
  5. I am too concerned about PrEP side effects
  6. I prefer to use condoms
  7. I don’t want people to think I am HIV-positive
  8. I don’t want to risk being stigmatized for taking PrEP
  9. Other (Please specify): ________________________

Current guidelines recommend that individuals on PrEP return to a medical provider every 3 months for HIV/STI testing, bloodwork, and a new 3-month prescription.

**PrEPComPresc**. Suppose that you were interested in getting a new prescription for PrEP – do you have or know of a medical provider that you would feel comfortable asking to prescribe it for you?

1. No, I definitely do not have or know of a provider
2. No, I probably do not have or know of a provider
3. Yes, I probably have or know of a provider
4. Yes, I definitely have or know of a provider

**PrEPComRec**. Suppose that you were interested in getting a new prescription for PrEP – where would you feel most comfortable receiving your PrEP-related medical care and prescriptions?

1. My primary care provider (my regular doctor)
2. A clinic specializing in HIV-related care (e.g., an HIV clinic)
3. A clinic specializing in sexual health (e.g., a Planned Parenthood, an STD clinic)
4. A clinic specializing in LGBT health care
5. Other (Please specify): ________________

**PrEPinject**. The Food and Drug Administration (FDA) has approved a new form of PrEP that involves getting an injection (i.e. from a needle) in the butt cheek every other month instead of a pill ever day. How interested would you be in getting injectable PrEP?

1. Not at all interested
2. A little interested
3. Somewhat interested
4. Very interested

## 2-1-1 Dosing

**[DISPLAY FOR ALL HIV NEGATIVE PARTICIPANTS]**

**prep211heard**. Have you ever heard of on-demand PrEP – also called event-based PrEP, event-driven PrEP, or PrEP 2-1-1?

1. No, I’ve never heard of it
2. I’ve heard of it, but don’t really know much about it
3. I know a little about it
4. I know a lot about it

[PAGE BREAK]

On-demand PrEP (also called Event Based Dosing or PrEP 2-1-1) is a non-daily PrEP regimen in which people:

- Take two PrEP pills 2-24 hours before they have sex
- Take one PrEP pill 24 hours later
- Take one more PrEP pill 24 hours after that
- Continue to take PrEP every 24 hours until they have 2 sex-free days

On-demand PrEP has been demonstrated in clinical trial to be as effective as daily oral PrEP as long as people are able to take it correctly (according to the schedule above). On-demand PrEP is currently being used in Europe, and some providers are recommending it here in the U.S. PrEP has not been FDA approved for on-demand use, and there are not currently CDC guidelines for it use.

For the rest of this survey, we are going to call this type of PrEP “PrEP 2-1-1.”

| **prep211agree**. Please rate your agreement with the following statements: | Strongly Disagree | Disagree | Neither Agree or Disagree | Agree | Strongly Agree |
| --- | --- | --- | --- | --- | --- |
| **prep211agree**1. I would like to find out more about PrEP 2-1-1 |  |  |  |  |  |
| **prep211agree**2. I would be interested in using PrEP 2-1-1 |  |  |  |  |  |
| **prep211agree**3. PrEP 2-1-1 would be a good HIV prevention choice for me |  |  |  |  |  |
| **prep211agree**4. It would be difficult for me to use PrEP 2-1-1 |  |  |  |  |  |

**prep211used**. Have you ever used PrEP 2-1-1?

1. Yes
2. No

**prep211often**. If you were using PrEP 2-1-1, how often do you think you would use it (i.e., take the four-pill regimen)?

1. I would never use PrEP 2-1-1
2. A couple of times per year
3. Every other month
4. Once per month
5. Two times per month
6. Three times per month
7. Once per week

**prep211time**. The last time you had sex, how much “lead time” did you have before it happened? That is, how much time did you plan in advance before you had sex?

1. No time, it just happened and I wasn’t expecting it
2. 30 minutes
3. 1 hour
4. 2 hours
5. 3-6 hours
6. 7-12 hours
7. 13-24 hours
8. More than 1 day

**prep211antibio**. If you were using PrEP 2-1-1, would you be willing to take a dose of antibiotics to prevent sexually transmitted infections at the same time?

1. Yes
2. No
3. Maybe

## Antibiotic and Dietary Supplement Use

**Diet1**. Have you taken antibiotics in the **past 6 months**?

1. Yes, I am taking antibiotics now
2. Yes, in the past week
3. Yes, between 1-4 weeks ago
4. Yes, between 1 and 2 months ago
5. Yes, between 2-3 months ago
6. Yes, between 3-6 months ago
7. No

**Diet2**. Have you taken probiotics (e.g., digestive health supplements) in the **past 6 months**?

1. Yes, I am taking probiotics now
2. Yes, in the past week
3. Yes, between 1 - 4 weeks ago
4. Yes, between 1 and 2 months ago
5. Yes, between 2-3 months ago
6. Yes, between 3-6 months ago
7. No
8. I don’t know what probiotics are

**Diet3**. Do you eat fermented products like yogurt?

1. Yes, in the past day
2. Yes, in the past week
3. Yes, between 1 - 4 weeks ago
4. Yes, between 1 and 2 months ago
5. Yes, between 2-3 months ago
6. Yes, between 3-6 months ago
7. No
8. I don’t know what fermented products are

**Diet4.** Do you follow a special diet or do you avoid certain foods (e.g., meat, fish, gluten, dairy)?

1. Yes (Please describe:) __________
2. No

**Diet5**. Have you experienced any vomiting, diarrhea, or other digestive disruptions recently?

1. Yes, in the past day
2. Yes, in the past week
3. Yes, between 1 - 4 weeks ago
4. Yes, between 1 and 2 months ago
5. Yes, between 2-3 months ago
6. Yes, between 3-6 months ago
7. No

**Diet6.** In the last year, have you taken antibiotics before or after sex to prevent (i.e., before you get diagnosed or have symptoms) getting a STI like chlamydia or syphilis?

1. Mo, I haven’t done this or have only taken antibiotics **after being diagnosed with an STI**
2. Yes, after having sex
3. Yes, before sex
4. Yes, before and after sex
5. I don’t know

Diet7. Do you have any of the following diets? **Please select all that apply.**

1. Vegetarian
2. Vegan
3. Gluten-free
4. Dairy-free
5. Ketogenic
6. Something else (Please specify):
7. Does not apply

## Food Insecurity

**FS1**. Since last (name of current month), did you ever cut the size of your meals or skip meals because there wasn’t enough money for food?

1. Yes
2. No
3. Don’t know

**[DISPLAY FS1a if FS1 = YES]**

**FS1a**. How often did this happen?

1. Only 1 or 2 months
2. Some months but not every month
3. Almost every month
4. Don’t know

**FS2**. Did you ever eat less than you felt you should because there wasn’t enough money for food?

1. Yes
2. No
3. Don’t know

**FS3**. Were you ever hungry but didn’t eat because there wasn’t enough money for food?

1. Yes
2. No
3. Don’t know

For the next 2 questions, please tell us how often each statement is true for you.

**FS4**. I couldn’t afford to eat balanced meals

1. Often true
2. Sometimes true
3. Never true
4. Don’t know

**FS5**. The food that I bought just didn’t last, and I didn’t have money to get more.

Never true

1. Often true
2. Sometimes true
3. Never true
4. Don’t know

**FS6.** In the past year, have you received SNAP (e.g.. EBT, “foodstamps”)?

1. Yes
2. No
3. I don’t know

## ASSIST

### ASSIST 1

In your life, which of the following substances have you ever used? For prescription medications, please report nonmedical use only.

|  | No | Yes |
| --- | --- | --- |
| ASSISTrev1_1. Marijuana (i.e., cannabis, pot, grass, hash, etc.) |  |  |
| ASSISTrev1_2. Crack (i.e., crack cocaine, rock, free base cocaine) |  |  |
| ASSISTrev1_3. Coke (i.e., cocaine, blow) |  |  |
| ASSISTrev1_4. Prescription stimulants (e.g., Ritalin, Concerta, Dexedrine, Adderall, diet pills, etc.) |  |  |
| ASSISTrev1_5. Methamphetamine (i.e., crystal meth, tina, ice, etc.) |  |  |
| ASSISTrev1_6. Inhalants (e.g., poppers, nitrous, glue, gas, paint thinner, etc.) |  |  |
| ASSISTrev1_7. Sedatives or sleeping pills (e.g., Valium, Ativan, Xanax, Klonopin, Librium, Rohypnol, etc.) |  |  |
| ASSISTrev1_8. GHB (i.e., gamma hydroxybutyrate, G) |  |  |
| ASSISTrev1_9. Molly (i.e. ecstasy, E, MDMA) |  |  |
| ASSISTrev1_10. Psychedelics (e.g., LSD, acid, mushrooms, 2C, PCP/angel dust) |  |  |
| ASSISTrev1_11. Ketamine (i.e., K, special K) |  |  |
| ASSISTrev1_12. Street opioids (e.g., heroin, opium, etc.) |  |  |
| ASSISTrev1_13. Prescription opioids (e.g., morphine, codeine, fentanyl, oxycodone/OxyContin/Percocet, hydrocodone/Vicodin, methadone, buprenorphine/Suboxone, etc.) |  |  |

### ASSIST 2

[**Display ASSIST2 if any ASSIST1=Yes]**

In the **past 3 months**, how often have you used the substances you mentioned?

**[DISPLAY FOR ITEMS ENDORSED IN ASSIST1]**

|  | Never | Once or twice | Monthly | Weekly | Daily or almost daily |
| --- | --- | --- | --- | --- | --- |
| ASSISTrev2_1. Marijuana (i.e., cannabis, pot, grass, hash, etc.) |  |  |  |  |  |
| ASSISTrev2_2. Crack (i.e., crack cocaine, rock, free base cocaine) |  |  |  |  |  |
| ASSISTrev2_3. Coke (i.e., cocaine, blow) |  |  |  |  |  |
| ASSISTrev2_4. Prescription stimulants (e.g., Ritalin, Concerta, Dexedrine, Adderall, diet pills, etc.) |  |  |  |  |  |
| ASSISTrev2_5. Methamphetamine (i.e., crystal meth, tina, ice, etc.) |  |  |  |  |  |
| ASSISTrev2_6. Inhalants (e.g., poppers, nitrous, glue, gas, paint thinner, etc.) |  |  |  |  |  |
| ASSISTrev2_7. Sedatives or sleeping pills (e.g., Valium, Ativan, Xanax, Klonopin, Librium, Rohypnol, etc.) |  |  |  |  |  |
| ASSISTrev2_8. GHB (i.e., gamma hydroxybutyrate, G) |  |  |  |  |  |
| ASSISTrev2_9. Molly (i.e. ecstasy, E, MDMA) |  |  |  |  |  |
| ASSISTrev2_10. Psychedelics (e.g., LSD, acid, mushrooms, 2C, PCP/angel dust) |  |  |  |  |  |
| ASSISTrev2_11. Ketamine (i.e., K, special K) |  |  |  |  |  |
| ASSISTrev2_12. Street opioids (e.g., heroin, opium, etc.) |  |  |  |  |  |
| ASSISTrev2_13. Prescription opioids (e.g., morphine, codeine, fentanyl, oxycodone/OxyContin/Percocet, hydrocodone/Vicodin, methadone, buprenorphine/Suboxone, etc.) |  |  |  |  |  |

**[DISPLAY ASSIST2Stim1 IF ASSISTrev2_4 =** **OTHER THAN NEVER]**

**ASSIST2Stim1**. You mentioned that you've used prescription stimulants (Ritalin, Concerta, Dexedrine, Adderall, diet pills, etc.) in the **past 3 months**. Is this a medication that you can buy in the store without a prescription (over the counter)?

1. Yes
2. No
3. Don’t know

**[DISPLAY ASSIST2Stim2 IF ASSIST2Stim1 = B or C]**

**ASSIST2Stim2**. Was it prescribed for you?

1. Yes
2. No
3. Don’t know

**[DISPLAY ASSIST2Stim3 AND ASSIST2Stim4 IF ASSIST2Stim2 = A]**

**ASSIST2Stim3.** Do you ever use **MORE** of your stimulant medication, that is, take a higher dosage, than is prescribed for you?

1. Yes
2. No
3. Don’t know

**ASSIST2Stim4.** Do you ever use your stimulant medication **MORE OFTEN**, that is, shorten the time between dosages, than is prescribed for you?

1. Yes
2. No
3. Don’t know

**[DISPLAY ASSIST2Sed IF OTHER THAN NEVER]**

**ASSIST2Sed1**. You mentioned that you've used sedatives or sleeping pills (Valium, Ativan, Xanax, Klonopin, Librium, Rohypnol, etc.) in the **past 3 months**. Is this a medication that you can buy in the store without a prescription (over the counter)?

1. Yes
2. No
3. Don’t know

**[DISPLAY ASSIST2Sed2 IF ASSIST2Sed1 = B or C]**

**ASSIST2Sed2**. Was it prescribed for you?

1. Yes
2. No
3. Don’t know

**[DISPLAY ASSIST2Sed3 AND ASSIST2Sed4 IF ASSIST2Sed2 = A]**

**ASSIST2Sed3**. Do you ever use MORE of your sedatives or sleeping pills, that is, take a higher dosage, than is prescribed for you?

1. Yes
2. No
3. Don’t know

**ASSIST2Sed4**. Do you ever use your sedatives or sleeping pills MORE OFTEN, that is, shorten the time between dosages, than is prescribed for you?

1. Yes
2. No
3. Don’t know

**[DISPLAY ASSIST2PrOpi1 IF OTHER THAN NEVER]**

**ASSIST2PrOpi1**. You mentioned that you've used prescription opioids (morphine, codeine, fentanyl, oxycodone [OxyContin, Percocet], hydrocodone [Vicodin], methadone, buprenorphine [Suboxone], etc.) in the **past 3 months**. Is this a medication that you can buy in the store without a prescription (over the counter)?

1. Yes
2. No
3. Don’t know

**[DISPLAY ASSIST2PrOpi2 IF ASSIST2PrOpi1 = B or C]**

**ASSIST2PrOpi2**. Was it prescribed for you?

1. Yes
2. No
3. Don’t know

**[DISPLAY ASSIST2PrOpi3 AND ASSIST2PrOpi4 IF ASSIST2PrOpi2 = A]**

**ASSIST2PrOpi3**. Do you ever use MORE of your opioid medication, that is, take a higher dosage, than is prescribed for you?

1. Yes
2. No
3. Don’t know

**ASSIST2PrOpi4**. Do you ever use your opioid medication MORE OFTEN, that is, shorten the time between dosages, than is prescribed for you?

1. Yes
2. No
3. Don’t know

### ASSIST 3

During the **past 3 months**, how often have you had a strong desire or urge to use the following?

**[DISPLAY FOR ITEMS ENDORSED IN ASSIST1]**

|  | Never | Once or twice | Monthly | Weekly | Daily or almost daily |
| --- | --- | --- | --- | --- | --- |
| ASSISTrev3_1. Marijuana (i.e., cannabis, pot, grass, hash, etc.) |  |  |  |  |  |
| ASSISTrev3_2. Crack (i.e., crack cocaine, rock, free base cocaine) |  |  |  |  |  |
| ASSISTrev3_3. Coke (i.e., cocaine, blow) |  |  |  |  |  |
| ASSISTrev3_4. Prescription stimulants (e.g., Ritalin, Concerta, Dexedrine, Adderall, diet pills, etc.) |  |  |  |  |  |
| ASSISTrev3_5. Methamphetamine (i.e., crystal meth, tina, ice, etc.) |  |  |  |  |  |
| ASSISTrev3_6. Inhalants (e.g., poppers, nitrous, glue, gas, paint thinner, etc.) |  |  |  |  |  |
| ASSISTrev3_7. Sedatives or sleeping pills (e.g., Valium, Ativan, Xanax, Klonopin, Librium, Rohypnol, etc.) |  |  |  |  |  |
| ASSISTrev3_8. GHB (i.e., gamma hydroxybutyrate, G) |  |  |  |  |  |
| ASSISTrev3_9. Molly (i.e. ecstasy, E, MDMA) |  |  |  |  |  |
| ASSISTrev3_10. Psychedelics (e.g., LSD, acid, mushrooms, 2C, PCP/angel dust) |  |  |  |  |  |
| ASSISTrev3_11. Ketamine (i.e., K, special K) |  |  |  |  |  |
| ASSISTrev3_12. Street opioids (e.g., heroin, opium, etc.) |  |  |  |  |  |
| ASSISTrev3_13. Prescription opioids (e.g., morphine, codeine, fentanyl, oxycodone/OxyContin/Percocet, hydrocodone/Vicodin, methadone, buprenorphine/Suboxone, etc.) |  |  |  |  |  |

### ASSIST 4

During the **past 3 months**, how often has your use of the following substances led to health, social, legal, or financial problems?

**[DISPLAY FOR ITEMS ENDORSED IN ASSIST1]**

|  | Never | Once or twice | Monthly | Weekly | Daily or almost daily |
| --- | --- | --- | --- | --- | --- |
| ASSISTrev4_1. Marijuana (i.e., cannabis, pot, grass, hash, etc.) |  |  |  |  |  |
| ASSISTrev4_2. Crack (i.e., crack cocaine, rock, free base cocaine) |  |  |  |  |  |
| ASSISTrev4_3. Coke (i.e., cocaine, blow) |  |  |  |  |  |
| ASSISTrev4_4. Prescription stimulants (e.g., Ritalin, Concerta, Dexedrine, Adderall, diet pills, etc.) |  |  |  |  |  |
| ASSISTrev4_5. Methamphetamine (i.e., crystal meth, tina, ice, etc.) |  |  |  |  |  |
| ASSISTrev4_6. Inhalants (e.g., poppers, nitrous, glue, gas, paint thinner, etc.) |  |  |  |  |  |
| ASSISTrev4_7. Sedatives or sleeping pills (e.g., Valium, Ativan, Xanax, Klonopin, Librium, Rohypnol, etc.) |  |  |  |  |  |
| ASSISTrev4_8. GHB (i.e., gamma hydroxybutyrate, G) |  |  |  |  |  |
| ASSISTrev4_9. Molly (i.e. ecstasy, E, MDMA) |  |  |  |  |  |
| ASSISTrev4_10. Psychedelics (e.g., LSD, acid, mushrooms, 2C, PCP/angel dust) |  |  |  |  |  |
| ASSISTrev4_11. Ketamine (i.e., K, special K) |  |  |  |  |  |
| ASSISTrev4_12. Street opioids (e.g., heroin, opium, etc.) |  |  |  |  |  |
| ASSISTrev4_13. Prescription opioids (e.g., morphine, codeine, fentanyl, oxycodone/OxyContin/Percocet, hydrocodone/Vicodin, methadone, buprenorphine/Suboxone, etc.) |  |  |  |  |  |

### ASSIST 5

During the **past 3 months**, how often have you failed to do what was normally expected of you because of your use of the following substances?

**[DISPLAY FOR ITEMS ENDORSED IN ASSIST1]**

|  | Never | Once or twice | Monthly | Weekly | Daily or almost daily |
| --- | --- | --- | --- | --- | --- |
| ASSISTrev5_1. Marijuana (i.e., cannabis, pot, grass, hash, etc.) |  |  |  |  |  |
| ASSISTrev5_2. Crack (i.e., crack cocaine, rock, free base cocaine) |  |  |  |  |  |
| ASSISTrev5_3. Coke (i.e., cocaine, blow) |  |  |  |  |  |
| ASSISTrev5_4. Prescription stimulants (e.g., Ritalin, Concerta, Dexedrine, Adderall, diet pills, etc.) |  |  |  |  |  |
| ASSISTrev5_5. Methamphetamine (i.e., crystal meth, tina, ice, etc.) |  |  |  |  |  |
| ASSISTrev5_6. Inhalants (e.g., poppers, nitrous, glue, gas, paint thinner, etc.) |  |  |  |  |  |
| ASSISTrev5_7. Sedatives or sleeping pills (e.g., Valium, Ativan, Xanax, Klonopin, Librium, Rohypnol, etc.) |  |  |  |  |  |
| ASSISTrev5_8. GHB (i.e., gamma hydroxybutyrate, G) |  |  |  |  |  |
| ASSISTrev5_9. Molly (i.e. ecstasy, E, MDMA) |  |  |  |  |  |
| ASSISTrev5_10. Psychedelics (e.g., LSD, acid, mushrooms, 2C, PCP/angel dust) |  |  |  |  |  |
| ASSISTrev5_11. Ketamine (i.e., K, special K) |  |  |  |  |  |
| ASSISTrev5_12. Street opioids (e.g., heroin, opium, etc.) |  |  |  |  |  |
| ASSISTrev5_13. Prescription opioids (e.g., morphine, codeine, fentanyl, oxycodone/OxyContin/Percocet, hydrocodone/Vicodin, methadone, buprenorphine/Suboxone, etc.) |  |  |  |  |  |

### ASSIST 6

Has a friend or relative or anyone else **ever** expressed concern about your use of the following substances?

**[DISPLAY FOR ITEMS ENDORSED IN ASSIST1]**

|  | No, never | Yes, in the past 3 months | Yes, but not in the past 3 months |
| --- | --- | --- | --- |
| ASSISTrev6_1. Marijuana (i.e., cannabis, pot, grass, hash, etc.) |  |  |  |
| ASSISTrev6_2. Crack (i.e., crack cocaine, rock, free base cocaine) |  |  |  |
| ASSISTrev6_3. Coke (i.e., cocaine, blow) |  |  |  |
| ASSISTrev6_4. Prescription stimulants (e.g., Ritalin, Concerta, Dexedrine, Adderall, diet pills, etc.) |  |  |  |
| ASSISTrev6_5. Methamphetamine (i.e., crystal meth, tina, ice, etc.) |  |  |  |
| ASSISTrev6_6. Inhalants (e.g., poppers, nitrous, glue, gas, paint thinner, etc.) |  |  |  |
| ASSISTrev6_7. Sedatives or sleeping pills (e.g., Valium, Ativan, Xanax, Klonopin, Librium, Rohypnol, etc.) |  |  |  |
| ASSISTrev6_8. GHB (i.e., gamma hydroxybutyrate, G) |  |  |  |
| ASSISTrev6_9. Molly (i.e. ecstasy, E, MDMA) |  |  |  |
| ASSISTrev6_10. Psychedelics (e.g., LSD, acid, mushrooms, 2C, PCP/angel dust) |  |  |  |
| ASSISTrev6_11. Ketamine (i.e., K, special K) |  |  |  |
| ASSISTrev6_12. Street opioids (e.g., heroin, opium, etc.) |  |  |  |
| ASSISTrev6_13. Prescription opioids (e.g., morphine, codeine, fentanyl, oxycodone/OxyContin/Percocet, hydrocodone/Vicodin, methadone, buprenorphine/Suboxone, etc.) |  |  |  |

### ASSIST 7

Have you **ever** tried and failed to control, cut down or stop using the following substances?

**[DISPLAY FOR ITEMS ENDORSED IN ASSIST1]**

|  | No, never | Yes, in the past 3 months | Yes, but not in the past 3 months |
| --- | --- | --- | --- |
| ASSISTrev7_1. Marijuana (i.e., cannabis, pot, grass, hash, etc.) |  |  |  |
| ASSISTrev7_2. Crack (i.e., crack cocaine, rock, free base cocaine) |  |  |  |
| ASSISTrev7_3. Coke (i.e., cocaine, blow) |  |  |  |
| ASSISTrev7_4. Prescription stimulants (e.g., Ritalin, Concerta, Dexedrine, Adderall, diet pills, etc.) |  |  |  |
| ASSISTrev7_5. Methamphetamine (i.e., crystal meth, tina, ice, etc.) |  |  |  |
| ASSISTrev7_6. Inhalants (e.g., poppers, nitrous, glue, gas, paint thinner, etc.) |  |  |  |
| ASSISTrev7_7. Sedatives or sleeping pills (e.g., Valium, Ativan, Xanax, Klonopin, Librium, Rohypnol, etc.) |  |  |  |
| ASSISTrev7_8. GHB (i.e., gamma hydroxybutyrate, G) |  |  |  |
| ASSISTrev7_9. Molly (i.e. ecstasy, E, MDMA) |  |  |  |
| ASSISTrev7_10. Psychedelics (e.g., LSD, acid, mushrooms, 2C, PCP/angel dust) |  |  |  |
| ASSISTrev7_11. Ketamine (i.e., K, special K) |  |  |  |
| ASSISTrev7_12. Street opioids (e.g., heroin, opium, etc.) |  |  |  |
| ASSISTrev7_13. Prescription opioids (e.g., morphine, codeine, fentanyl, oxycodone/OxyContin/Percocet, hydrocodone/Vicodin, methadone, buprenorphine/Suboxone, etc.) |  |  |  |

### ASSIST 8

**Assist8a**. Have you ever used any drug by injection (recreational on non-medical use only)?

1. Yes, in the **past 3 months**
2. Yes, but **not** in the past 3 months
3. No, never

**[DISPLAY Assist8b IF Assist8a = A]**

**Assist8b**. In the **past 3 months**, how often have you injected drugs (recreational or non-medical use only)?

1. Once per week or less
2. More than once per week

## PNP

**[DISPLAY THIS SECTION ONLY IF ASSISTrev2_2, ASSISTrev2_3, ASSISTrev2_4, ASSISTrev2_5, ASSISTrev2_8, or ASSISTrev2_9 ARE NOT “NEVER”]**

**PNPparty**. In the last three months, how often have you sought out people to "party" with (e.g., meth, Tina, GHB)?

1. Daily or almost daily
2. Weekly
3. Monthly
4. Once or twice
5. Never

**PNPspend**. In an average month, how much do you spend on stimulants like meth and coke?

1. Zero dollars
2. 1 to 50 dollars
3. 51 to 100 dollars
4. 101 to 200 dollars
5. 201 to 300 dollars
6. 301 to 400 dollars
7. More than 401 dollars

**[DISPLAY MethHouse IF ASSISTrev2_5 IS NOT NEVER]**

**MethHouse**. Do you keep meth in the house?

1. Yes
2. No
3. Not applicable

**[DISPLAY CokeHouse IF ASSISTrev2_3 IS NOT NEVER]**

**CokeHouse.** Do you keep coke in the house?

1. Yes
2. No
3. Not applicable

## Substance Use Networks

**NetworkNames.** Please list 3 people who you consider to be your close contacts, that is, people who you talk to or hang out with often. Close contacts This can include friends, romantic partners, sexual partners, or even family members. It's ok if you don't hang out with them that often, just put the three people you hang out with most. **Please put their first name or a nickname.**

1. Person 1: [text entry]
2. Person 2: [text entry]
3. Person 3: [text entry]

**[PLEASE DISPLAY THE FOLLOWING FOR EACH OF THE THREE PEOPLE ENTERED ABOVE]**

**NetworkAgeP#.** What is [PERSON#]’s age?

1. [Numeric entry validated to < 99]

**NetworkRaceP#.** What is [PERSON#]’s ethnic or racial identity? **Please select all that apply.**

1. Black or African American
2. East Asian
3. Latino, Latinx, or Latin American
4. Middle Eastern or North African
5. Native American, American Indian, or Alaska Native
6. Native Hawaiian or other Pacific Islander
7. South Asian
8. White or European American
9. Multiracial or Multiethnic
10. I don’t know
11. Something else (Please specify):

**NetworkGenderP#.** What is <PERSON#>’s gender identity?

1. Cis man
2. Cis woman
3. Trans man
4. Trans woman
5. Non-binary, genderqueer, genderfluid
6. Agender
7. Questioning
8. Something else (Please specify):

**NetworkRelationshipP#.** What is your relationship with <PERSON#>? **Please select all that apply.**

1. Immediate family (mother, father, sibling)
2. Extended/other family
3. Main partner or significant other
4. Casual or other sex partner
5. LGBTQ-identified close friend, someone you see or talk to regularly
6. Non-LGBTQ-identified close friend, someone you see or talk to regularly
7. Acquaintance, someone you know, but don’t see or talk to often
8. Coworker
9. Something else (Please specify):

**Network_drink_freqP#**. In the **past 30 days**, how many days have you had drinks containing alcohol with <PERSON#>?

1. [Numeric entry, limit 0 - 31]

**Network_drunkP#**. In the **past 30 days**, how many days have you gotten drunk with <PERSON#>?

1. [Numeric entry, limit 0 - 31]

**Network_substanceP#**. In the **past 30 days**, have you used any of the following substances with <PERSON#>? **Please select all that apply.**

1. Marijuana (i.e., cannabis, pot, grass, hash, etc.)
2. Crack (i.e., crack cocaine, rock, free base cocaine)
3. Coke (i.e., cocaine, blow)
4. Methamphetamine (i.e., crystal meth, tina, ice, etc.)
5. Inhalants (e.g., poppers, nitrous, glue, gas, paint thinner, etc.)
6. GHB (i.e., G, gamma hydroxybutyrate)
7. Molly (i.e, ecstasy, E, MDMA)
8. Psychedelics (e.g., LSD, acid, mushrooms, 2C, PCP, angel dust, etc.)
9. Ketamine (K, special K)
10. Street opioids (e.g., heroin, opium, etc.)
11. Prescription stimulants (e.g., Ritalin, Concerta, Dexedrine, Adderall, diet pills, etc.)
12. Prescription sedatives or sleeping pills (Valium, Ativan, Xanax, Klonopin, Librium, Rohypnol, etc.)
13. Prescription opioids (e.g., morphine, codeine, fentanyl, oxycodone/OxyContin/Percocet, hydrocodone/Vicodin, methadone, buprenorphine/Suboxone, etc.)
14. We have not used any of these substances [EXCLUSIVE]

## AUDIT-10 Alcohol

The following questions will be about alcohol use and a “standard drink.” A standard drink is one 12oz can of beer, one glass of wine, or the equivalent of one shot of hard alcohol.

**AUDIT1**. How often do you have a drink containing alcohol?

1. Never
2. Monthly or less
3. 2-4 times a month
4. 2-3 times a week
5. 4 or more times a week

[**IF AUDIT1=A SKIP to end of block]**

**AUDIT2**. How many drinks containing alcohol do you have on a typical day when you are drinking?

1. 1 or 2
2. 2 or 4
3. 5 or 6
4. 7 to 9
5. 10 or more

**AUDIT3**. How often do you have **six or more** drinks on one occasion?

1. Never
2. Less than monthly
3. Monthly
4. Weekly
5. Daily or almost daily

**AUDIT4**. How often during the **past year** have you found that you were not able to stop drinking once you had started?

1. Never
2. Less than monthly
3. Monthly
4. Weekly
5. Daily or almost daily

**AUDIT5**. How often during the **past year** have you failed to do what was normally expected of you because of drinking?

1. Never
2. Less than monthly
3. Monthly
4. Weekly
5. Daily or almost daily

**AUDIT6**. How often during the **past year** have you needed a first drink in the morning to get yourself going after a heavy drinking session?

1. Never
2. Less than monthly
3. Monthly
4. Weekly
5. Daily or almost daily

**AUDIT7**. How often during the **past year** have you had a feeling of guilt or remorse after drinking?

1. Never
2. Less than monthly
3. Monthly
4. Weekly
5. Daily or almost daily

**AUDIT8**. How often during the **past year** have you been unable to remember what happened the night before because of your drinking?

1. Never
2. Less than monthly
3. Monthly
4. Weekly
5. Daily or almost daily

**AUDIT9**. Have you or someone else been injured because of your drinking?

1. No
2. Yes, but not in the past year
3. Yes, during the past year

**AUDIT10**. Has a relative, friend, doctor, or other health care worker been concerned about your drinking or suggested you cut down?

1. No
2. Yes, but not in the past year
3. Yes, during the past year

## Self Perceived Need for Treatment

**[DISPLAY ONLY FOR THOSE WHO ANSWER AUDIT1 AS OTHER THAN NEVER]**

**AlchBothered.** In the past 30 days, how troubled or bothered have you been by problems that you have experienced with alcohol?

1. Not at all
2. Slightly
3. Moderately
4. Considerably
5. Extremely
6. Not applicable

**DrugBothered.** In the past 30 days, how troubled or bothered have you been by problems that you have experienced with drugs?

1. Not at all
2. Slightly
3. Moderately
4. Considerably
5. Extremely
6. Not applicable

**AlchTreat.** Right now, how important is it for you to receive treatment for problems you have experienced with alcohol?

1. Not at all
2. Slightly
3. Moderately
4. Considerably
5. Extremely
6. Not applicable

**DrugTreat.** Right now, how important is it for you to receive treatment for problems you have experienced with drugs?

1. Not at all
2. Slightly
3. Moderately
4. Considerably
5. Extremely
6. Not applicable

## CES-D 10 Depression

| During the **past week**: | Rarely or none of the time  (less than 1 day) | Some or a little of the time  (1-2 days) | Occasionally or a moderate amount of time  (3-4 days) | Most or all of the time  (5-7 days) |
| --- | --- | --- | --- | --- |
| **CESD1.** I was bothered by things that usually don’t bother me |  |  |  |  |
| **CESD2.** I had trouble keeping my mind on what I was doing |  |  |  |  |
| **CESD3.** I felt depressed. |  |  |  |  |
| **CESD4.** I felt that everything I did was an effort |  |  |  |  |
| **CESD5.** I felt hopeful about the future |  |  |  |  |
| **CESD6.** I felt fearful |  |  |  |  |
| **CESD7.** My sleep was restless |  |  |  |  |
| **CESD8.** I was happy |  |  |  |  |
| **CESD9.** I felt lonely |  |  |  |  |
| **CESD10.** I could not get “going” |  |  |  |  |

## GSRS

**GSRS1.** Have you been bothered by PAIN OR DISCOMFORT IN YOUR UPPER ABDOMEN OR THE PIT OF YOUR STOMACH during the past week?

1. No discomfort at all
2. Minor discomfort
3. Mild discomfort
4. Moderate discomfort
5. Moderately severe discomfort
6. Severe discomfort
7. Very severe discomfort

**GSRS2.** Have you been bothered by HEARTBURN during the past week? (By heartburn we mean an unpleasant stinging or burning sensation in the chest.)

- 1. No discomfort at all
  2. Minor discomfort
  3. Mild discomfort
  4. Moderate discomfort
  5. Moderately severe discomfort
  6. Severe discomfort
  7. Very severe discomfort

**GSRS3**. Have you been bothered by ACID REFLUX during the past week? (By acid reflux we mean the sensation of regurgitating small quantities of acid or flow of sour or bitter fluid from the stomach up to the throat.)

1. No discomfort at all
2. Minor discomfort
3. Mild discomfort
4. Moderate discomfort
5. Moderately severe discomfort
6. Severe discomfort
7. Very severe discomfort

4. Have you been bothered by HUNGER PAINS in the stomach during the past week? (This hollow feeling in the stomach is associated with the need to eat between meals.)

• No discomfort at all

• Minor discomfort

• Mild discomfort

• Moderate discomfort

• Moderately severe discomfort

• Severe discomfort

• Very severe discomfort

5. Have you been bothered by NAUSEA during the past week? (By nausea we mean a feeling of sickness that may lead to retching and vomiting.)

• No discomfort at all

• Minor discomfort

• Mild discomfort

• Moderate discomfort

• Moderately severe discomfort

• Severe discomfort

• Very severe discomfort

6. Have you been bothered by RUMBLING in your stomach during the past week? (Rumbling refers to vibrations or noise in the stomach.)

• No discomfort at all

• Minor discomfort

• Mild discomfort

• Moderate discomfort

• Moderately severe discomfort

• Severe discomfort

• Very severe discomfort

7. Has your stomach felt BLOATED during the past week? (Feeling bloated refers to swelling often associated with a sensation of gas or air in the stomach.)

• No discomfort at all

• Minor discomfort

• Mild discomfort

• Moderate discomfort

• Moderately severe discomfort

• Severe discomfort

• Very severe discomfort

8. Have you been bothered by BELCHING during the past week? (Belching refers to the release of wind from the stomach via the mouth, often associated with easing a bloated feeling.)

• No discomfort at all

• Minor discomfort

• Mild discomfort

• Moderate discomfort

• Moderately severe discomfort

• Severe discomfort

• Very severe discomfort

9. Have you been bothered by BREAKING WIND during the past week? (Breaking wind refers to the need to release air or gas from the bowel, often associated with easing a bloated feeling.)

• No discomfort at all

• Minor discomfort

• Mild discomfort

• Moderate discomfort

• Moderately severe discomfort

• Severe discomfort

• Very severe discomfort

10. Have you been bothered by CONSTIPATION during the past week? (Constipation refers to a reduced ability to empty the bowels.)

• No discomfort at all

• Minor discomfort

• Mild discomfort

• Moderate discomfort

• Moderately severe discomfort

• Severe discomfort

• Very severe discomfort

11. Have you been bothered by DIARRHOEA during the past week? (Diarrhoea refers to a too frequent emptying of the bowels.)

• No discomfort at all

• Minor discomfort

• Mild discomfort

• Moderate discomfort

• Moderately severe discomfort

• Severe discomfort

• Very severe discomfort

12. Have you been bothered by LOOSE STOOLS during the past week? (If your stools (motions) have been alternately hard and loose, this question only refers to the extent you have been bothered by the stools being loose.)

• No discomfort at all

• Minor discomfort

• Mild discomfort

• Moderate discomfort

• Moderately severe discomfort

• Severe discomfort

• Very severe discomfort

13. Have you been bothered by HARD STOOLS during the past week? (If your stools (motions) have been alternately hard and loose, this question only refers to the extent you have been bothered by the stools being hard.)

• No discomfort at all

• Minor discomfort

• Mild discomfort

• Moderate discomfort

• Moderately severe discomfort

• Severe discomfort

• Very severe discomfort

14. Have you been bothered by an URGENT NEED TO HAVE A BOWEL MOVEMENT during the past week? (This urgent need to go to the toilet is often associated with a feeling that you are not in full control.)

• No discomfort at all

• Minor discomfort

• Mild discomfort

• Moderate discomfort

• Moderately severe discomfort

• Severe discomfort

• Very severe discomfort

15. When going to the toilet during the past week, have you had the SENSATION OF NOT COMPLETELY EMPTYING THE BOWELS? (This feeling of incomplete emptying means that you still feel a need to pass your motions despite having exerted yourself to do so.)

• No discomfort at all

• Minor discomfort

• Mild discomfort

• Moderate discomfort

• Moderately severe discomfort

• Severe discomfort

• Very severe discomfort

## GAD-7 Anxiety

| In the **past 2 weeks**, how often have you been bothered by the following? | Not at all | Several days | More than half the days | Nearly every day |
| --- | --- | --- | --- | --- |
| **GAD-7_1.** Feeling nervous, anxious, or on edge |  |  |  |  |
| **GAD-7_2.** Not being able to stop or control worrying |  |  |  |  |
| **GAD-7_3.** Worrying too much about different things |  |  |  |  |
| **GAD-7_4.** Trouble relaxing |  |  |  |  |
| **GAD-7_5.** Being so restless that it's hard to sit still |  |  |  |  |
| **GAD-7_6.** Becoming easily annoyed or irritable |  |  |  |  |
| **GAD-7_7.** Feeling afraid as if something awful might happen |  |  |  |  |

## Hypersexual Disorder Inventory

| During the **past 6 months**, how often have each of the following statements been true of you? | Never true | Rarely true | Sometimes true | Often true | Almost always true |
| --- | --- | --- | --- | --- | --- |
| **HSDI1.** I have spent a great amount of time consumed by sexual fantasies and urges as well as planning for and engaging in sexual behavior |  |  |  |  |  |
| **HSDI2.** I have used sexual fantasies and sexual behavior to cope with difficult feelings (e.g., worry, sadness, boredom, frustration, guilt, or shame) |  |  |  |  |  |
| **HSDI3.** I have used sexual fantasies and sexual behavior to avoid, put off, or cope with stresses and other difficult problems or responsibilities in my life |  |  |  |  |  |
| **HSDI4.** I have tried to reduce or control the frequency of sexual fantasies, urges, and behavior but I have not been very successful |  |  |  |  |  |
| **HSDI5.** I have continued to engage in risky sexual behavior that could or has caused injury, illness, or emotional damage to myself, my sexual partner(s), or a significant relationship |  |  |  |  |  |
| **HSDI6.** Frequent and intense sexual fantasies, urges and behavior have made me feel very upset or bad about myself (e.g., feelings of shame, guilt, sadness, worry, or disgust) or I tried to keep my sexual behavior a secret |  |  |  |  |  |
| **HSDI7.** Frequent and intense sexual fantasies, urges and behavior have caused significant problems for me in personal, social, work, or other important areas of my life |  |  |  |  |  |

## Main and Other Partners

**MainPartner**. Are you currently in a relationship with someone to whom you feel committed above anyone else and with whom you have had a sexual relationship?

1. Yes
2. No

**Polyamorous**. Are you currently in a polyamorous relationship (a committed relationship with more than one person, a thrupple)?

1. Yes
2. No

**[IF Polyamorous = yes then ask PolyMain and PolySecondary]**

**PolyMain.** How many **main** poly partners do you have?

1. [0-90]

**PolySecondary.** How many **secondary** poly partners do you have?

1. [0-90]

**[IF MainPartner = yes, ASK OutsideSex THROUGH PartnerPrEP]**

**OutsideSex.** How do you and your main partner handle outside sex?

1. Monogamous - We only have sex with each other
2. Open for them - Only they have sex with outside partners
3. Open for me - Only I have sex with outside partners
4. Open, but never together- My partner and I have sex with outside partners, but only separately
5. Only together - We only have sex with outside partners as a couple (3 way)
6. Fully open - We both have sex with outside partners, separately and together
7. It’s complicated - I have sex with others, but don’t know about my partner

**PartnerAge.** What is your main partner’s age?

1. [Age Entry]

**PartnerGenderOth.** What is your main partner’s gender identity?

1. Cis man
2. Cis woman
3. Trans man
4. Trans woman
5. Non-binary, genderqueer, genderfluid
6. Agender
7. Questioning
8. Something else (Please specify):

**PartnerEthnicity.** What is your main partner’s race or ethnicity? **Please select all that apply.**

1. Black or African American
2. East Asian
3. Latino, Latinx, or Latin American
4. Middle Eastern or North African
5. Native American, American Indian, or Alaska Native
6. Native Hawaiian or other Pacific Islander
7. South Asian
8. White or European American
9. Multiracial or Multiethnic
10. Something else (Please specify):

**RelationLength.** How long have you been with your main partner?

1. Less than a month
2. 1 to 3 months
3. 4 to 6 months
4. 6 months to 1 year
5. 1 to 5 years
6. 5 to 10 years
7. 10 to 20 years
8. More than 20 years

**UAVIMain**. How many times have you had anal/vaginal/front hole sex without a condom with your main partner in the **last 3 months (since Month, Year)**?

1. [0-989]

**PartnerStatus**. What is your main partner’s HIV status?

1. My partner told me they are HIV-positive
2. I think my partner is HIV-positive
3. I don’t know my partner’s HIV status
4. I think my partner is HIV-negative
5. My partner told me they are HIV-negative

**[IF PartnerStatus = A, B, ASK PartnerVL]**

**PartnerVL**. Is your main partner’s HIV viral load undetectable?

1. Yes
2. No
3. I don’t know

**[IF PartnerStatus = C, D, E, ASK PartnerPrEP]**

**PartnerPrEP.** Is your main partner on pre-exposure prophylaxis (PrEP)?

1. Yes
2. No
3. I don’t know

## Felt Stigma

These questions are about things that might happen to you because of **who you are**. This includes both **how you describe or see yourself** and **how others might describe or see you**. For example, your skin color, ancestry, nationality, religion, gender, sexuality, occupation, drug use status, age, weight, disability or mental health status, and income.

| Please rate your agreement with the following statements. Because of who I am… | Strongly Disagree | Disagree | Neither Agree nor Disagree | Agree | Strongly Agree |
| --- | --- | --- | --- | --- | --- |
| **Stigma1**. I worry that a doctor, nurse, or other health care provider might treat me poorly. |  |  |  |  |  |
| **Stigma2**. I worry about being treated unfairly by a teacher, supervisor, or employer. |  |  |  |  |  |
| **Stigma3**. I worry about being harassed or stopped by police or security. |  |  |  |  |  |

| Please rate your agreement with the following statements. | Strongly Disagree | Disagree | Neither Agree nor Disagree | Agree | Strongly Agree |
| --- | --- | --- | --- | --- | --- |
| **Racism1**. Doctors treat people of color and white people the same. |  |  |  |  |  |
| **Racism2**. Racial discrimination in a healthcare provider’s office is common. |  |  |  |  |  |
| **Racism3**. With most doctors, people of color and whites receive the same kind of care. |  |  |  |  |  |
| **Racism4.** People of color can receive the care they want as equally as white people can**.** |  |  |  |  |  |

| Please rate your agreement with the following statements. | Strongly Disagree | Disagree | Neither Agree nor Disagree | Agree | Strongly Agree |
| --- | --- | --- | --- | --- | --- |
| **MedMistrust1**. Patients have sometimes been deceived or misled at hospitals. |  |  |  |  |  |
| **MedMistrust2**. Hospitals often want to know more about your personal affairs or business than they really need to know. |  |  |  |  |  |
| **MedMistrust3**. Rich patients receive better care at hospitals than poor patients do. |  |  |  |  |  |
| **MedMistrust4.** Male patients receive better care at hospitals than female patients do. |  |  |  |  |  |
| **MedMistrust5.** Hospitals have sometimes done harmful experiments on patients without their knowledge. |  |  |  |  |  |

| Please rate your agreement with the following statements. | Strongly Disagree | Disagree | Neither Agree nor Disagree | Agree | Strongly Agree |
| --- | --- | --- | --- | --- | --- |
| **InDI-A1.** Because of who I am, I might have trouble finding or keeping a job. |  |  |  |  |  |
| **InDI-A2.** Because of who I am, I might have trouble getting an apartment or house. |  |  |  |  |  |
| **InDI-A3.** I worry about being treated unfairly by a teacher, supervisor, or employer. |  |  |  |  |  |
| **InDI-A4.** I may be denied a bank account, loan, or mortgage because of who I am. |  |  |  |  |  |
| **InDI-A5.** I worry about being harassed or stopped by police or security. |  |  |  |  |  |
| **InDI-A6.** Because of who I am, people might try to attack me physically. |  |  |  |  |  |
| **InDI-A7.** I expect to be pointed at, called named, or harassed when in public. |  |  |  |  |  |
| **InDI-A8.** I fear that I will have a hard time finding a friendship or romance because of who I am. |  |  |  |  |  |
| **InDI-A9.** Because of who I am, a doctor, nurse or other healthcare provider might treat me poorly. |  |  |  |  |  |

## Internalized Homophobia

**[DISPLAY IF GENDER=CISMEN]**

| How much do you disagree or agree with the following statements? | Strongly Disagree | Disagree | Neither Disagree or Agree | Agree | Strongly Agree | Decline to answer |
| --- | --- | --- | --- | --- | --- | --- |
| **IHS1.** I wish I wasn’t sexually attracted to men. |  |  |  |  |  |  |
| **IHS2.** I have tried to stop being sexually attracted to men in general. |  |  |  |  |  |  |
| **IHS3.** If someone offered me the chance to be completely heterosexual, I would accept the chance. |  |  |  |  |  |  |
| **IHS4.** I feel like being sexually attracted to men is a personal shortcoming for me. |  |  |  |  |  |  |
| **IHS5**. I would like to get professional help in order to change my sexual attraction to men. |  |  |  |  |  |  |

## Sexual Minority Stress

| There are important differences in suicide presentation and risk among ethnic and sexual minority groups, with those differences mainly stemming from cultural background. It is important to understand how a person’s culture influences their beliefs about the world and themselves. These questions ask about beliefs about your sexual orientation that may cause or increase stress for you. | Strongly disagree | Moderately disagree | Slightly disagree | Slightly agree | Moderately agree | Strongly agree | Decline to answer |
| --- | --- | --- | --- | --- | --- | --- | --- |
| **CARS1**. The decision to hide or reveal my sexual orientation to others causes me significant distress. |  |  |  |  |  |  |  |
| **CARS2**. Because of my sexual orientation, no one understands my pain or distress. |  |  |  |  |  |  |  |
| **CARS3**. I was rejected by a family member or friend after telling them my sexual orientation. |  |  |  |  |  |  |  |
| **CARS4**. I feel confused or conflicted by my sexual orientation. |  |  |  |  |  |  |  |
| **CARS5**. I feel comfortable revealing my sexual attraction and/or behavior. |  |  |  |  |  |  |  |
| **CARS6**. People treat me unfairly because of my sexual identity. |  |  |  |  |  |  |  |
| **CARS7**. At times, I feel I stick out because of my sexual attractions. |  |  |  |  |  |  |  |
| **CARS8.** Stereotypes about gay and bisexual people hurt my self-esteem or the way I see myself. |  |  |  |  |  |  |  |
| **CARS9.** I believe the world is a dangerous place to be gay or bisexual. |  |  |  |  |  |  |  |

## Substance Use Stigma

**[DISPLAY THIS SECTION IF ANY ASSISTrev2 = YES]**

The following questions ask about your alcohol and/or drug use history this includes any past or current experiences using alcohol and/or drugs. Please think about each question and select your answer. The first group of questions asks about how people have treated you **in the past** because of alcohol and/or drug use history. The second group of questions asks about how people will treat you **in the future** because of your alcohol and/or drug use history.

| How often have people treated you this way in the past because of your alcohol and/or drug use history? | Never | Not often | Somewhat often | Often | Very often |
| --- | --- | --- | --- | --- | --- |
| **EnSubStig1.** Family members have thought that I cannot be trusted. |  |  |  |  |  |
| **EnSubStig2.** Family members have looked down on me. |  |  |  |  |  |
| **EnSubStig3.** Family members have treated me differently. |  |  |  |  |  |
| **EnSubStig4.** Healthcare workers have not listened to my concerns. |  |  |  |  |  |
| **EnSubStig5.** Healthcare workers have thought that I’m pill shopping or trying to con them into giving me prescription medications to get high or sell. |  |  |  |  |  |
| **EnSubStig6.** Healthcare workers have given me poor care. |  |  |  |  |  |

| How likely is it that people will treat you in the following ways in the future because of your alcohol and/or drug use history? | Very Unlikely | Unlikely | Neither unlikely or likely | Likely | Very likely |
| --- | --- | --- | --- | --- | --- |
| **AnSubStig1.** Family members will think that I cannot be trusted. |  |  |  |  |  |
| **AnSubStig2.** Family members will look down on me. |  |  |  |  |  |
| **AnSubStig3.** Family members will treat me differently. |  |  |  |  |  |
| **AnSubStig4.** Healthcare workers will not listen to my concerns. |  |  |  |  |  |
| **AnSubStig5.** Healthcare workers will think that I’m pill shopping or trying to con them into giving me prescription medications to get high or sell. |  |  |  |  |  |
| **AnSubStig6.** Healthcare workers will give me poor care. |  |  |  |  |  |

| How do you feel about your alcohol and/or drug use history? | Strongly disagree | Disagree | Neither agree or disagree | Agree | Strongly agree |
| --- | --- | --- | --- | --- | --- |
| **InSubStig1.** Having used alcohol/drugs makes me feel like I’m a bad person. |  |  |  |  |  |
| **InSubStig2.** I feel I’m not as good as others because I use alcohol/drugs. |  |  |  |  |  |
| **InSubStig3.** I feel ashamed of having used alcohol/drugs. |  |  |  |  |  |
| **InSubStig4.** I think less of myself because I used alcohol/drugs. |  |  |  |  |  |
| **InSubStig5.** Having used alcohol/drugs makes me feel unclean. |  |  |  |  |  |
| **InSubStig6.** Having used alcohol/drugs is disgusting to me. |  |  |  |  |  |

## Injection Drug Use

**Inject.** In the **past year**, have you **injected** any drugs recreationally?

1. Yes
2. No

**[IF Inject = No SKIP TO END OF SECTION]**

**InjectWhat.** In the **past year**, what drugs have you **injected** recreationally? **Please select all that apply.**

1. Methamphetamine (speed, crystal meth, tina, ice, etc.)
2. Cocaine or crack (coke, blow, crack, etc.)
3. Street opioids (heroin, opium, etc.)
4. Prescription opioids (morphine, codeine, fentanyl, oxycodone/OxyContin/Percocet, hydrocodone/Vicodin, methadone, buprenorphine/Suboxone, etc.)
5. Ketamine (K, Special K)
6. Something else (Please specify):

**InjectAdmin**. In the **past year**, when you injected, did you do it yourself or did someone do it for you (admin)?

1. I did it myself
2. Someone else did it for me
3. Both (I did it myself and someone else did it for me)

**InjectShare**. In the **past year**, have you reused or shared an injection needle with someone else? **Please select all that apply.**

1. I’ve shared needles with someone else
2. I have reused needles I have previously used
3. Neither (I have always used a new needle) [exclusive]
4. I don’t know [exclusive]

**InjectSource**. In the **past year**, where did you get your syringes (needles) from? **Please select all that apply.**

1. From someone else
2. Syringe service program (e.g., needle exchange)
3. Ordered online (e.g., Amazon)
4. Drug store (e.g., CVS)
5. Something else (Please specify): _________

**InjectWound**. In the past year, have you developed a wound (i.e., abscess) as a result of injecting drugs?

1. Yes
2. No
3. I don’t know, don’t remember

**InjectOnset**. How old were you the first time you injected drugs for recreational use?

1. [VALIDATED NUMBER ENTRY]

**Meth3Mdays**. In the **last 3 months**, about how many days have you used methamphetamine (i.e. crystal meth, tina, ice, etc.)?

1. I have not used methamphetamine in the last three months
2. 1-5 days
3. 6-10 days
4. 11-20 days
5. 21-30 days
6. 31 or more days

## Insurance

**PrimCare**. Do you have someone you consider your primary care provider (a doctor)?

1. Yes
2. No

**[DISPLAY PrimCareSexMen IF PrimCare = YES]**

**PrimCareSexMen**. Do they know that you have sex with men?

1. Yes
2. No

**InsuranceType.** What type of health insurance do you currently have?

1. I do not have health insurance
2. Private Insurance from an employer, union or school (e.g. Blue Cross/Blue Shield, United, Kaiser)
3. A health insurance plan that you signed up for through a health insurance marketplace (e.g. HealthCare.gov, NY State of Health, Covered California, an “Obamacare”/ACA plan)
4. A health insurance plan that you bought directly from an insurance company.
5. Medicaid
6. Medicare
7. Some other government program (e.g. TriCare, tribal health care)
8. Something else (Please specify): ___________
9. Don’t know/Not sure

**[IF InsuranceType = don’t have or don’t know SKIP TO CareAvoid]**

**PolicyHolder.** Is your insurance under your own name, i.e. are you the policy holder?

1. Yes
2. No, I'm insured under my parents'/guardian's policy
3. No, I'm insured under my spouse or domestic partner policy
4. Don’t know/Not sure

**[IF PolicyHolder = no or don’t know DISPLAY PrivacyImpact]**

**PrivacyImpact.** Does the possibility that someone might see those documents impact your decision about getting healthcare (e.g., seeing a medical provider)?

1. Not at all
2. A little bit
3. Somewhat
4. A lot
5. I'm not sure

**Premium.** About how much do you pay every month in premiums for your main health insurance plan? This includes money deducted from a paycheck, or social security check, as well as money you pay directly to the insurance company. If you receive government financial assistance, this is just the amount that you are responsible for paying.

1. I don’t pay an insurance premium
2. $40 or less
3. $40 to 125
4. $126 to $250
5. $251 to $400
6. $401 to $500
7. $501 to $700
8. $700 or more
9. Don’t know/Not sure

**[DISPLAY Premfam IF Premium = a number]**

**Premfam.** Is this premium amount just for you (i.e., coverage for a single person), or is it for a family plan?

1. Own coverage only/Individual plan
2. Family plan (includes plans that cover spouse, children and any others)
3. Don’t know/Not sure

**Deduct.** If you have an annual deductible, **what is your deductible?**

A deductible is the amount you have to pay before your insurance plan will start paying any part of your medical bills. This is different from a co-pay, which is payment for a doctor visit or other medical service.

1. $100 or less
2. $100 to $200
3. $201 to $500
4. $501 to $1,000
5. $1,001 to $3,000
6. $3,001 to $5,000
7. $5,001 or more
8. I don’t have a deductible
9. Don’t know/Not sure

**MedSpend**. About how much have you spent “out of pocket” for medical expenses in the past 12 months? Your best guess is fine.

“Out of pocket” means **any** amount of money you have to pay toward a medical service that is NOT covered by any insurance or special assistance you might have.

1. $100 or less
2. $100 to $200
3. $201 to $500
4. $501 to $1,000
5. $1,001 to $3,000
6. $3,001 to $5,000
7. $5,001 or more
8. I did not pay “out of pocket”
9. Don’t know

**Careavoid.** In **past year,** because of cost have you been unable to do any of the following? **Please select all that apply.**

1. Fill a prescription for medicine
2. Receive a recommended test, treatment, or follow-up appointment
3. Visit the doctor despite having a medical problem
4. See a specialist when you or your doctor thought you needed one
5. Not applicable

**Medbills.** In the past **two years**, have any of the following happened because of medical bills? **Please select all that apply.**

1. Been unable to pay for basic necessities like food, heat or rent
2. Used up all your savings
3. Taken out a mortgage against your home or taken out a loan
4. Taken on credit card debt
5. Had to declare bankruptcy
6. Delayed education or career plans
7. Received a lower credit rating
8. Not applicable [EXCLUSIVE]

## PTSD-5

Sometimes things happen to people that are unusually or especially frightening, horrible, or traumatic. For example:

- a serious accident or fire
- a physical or sexual assault or abuse
- an earthquake or flood
- a war
- seeing someone be killed or seriously injured
- having a loved one die through homicide or suicide.

**PTSD1**. Have you ever experienced this kind of event?

1. Yes
2. No
3. Decline to answer

**[IF PTSD1 = No OR Decline to answer SKIP TO THE END OF THE BLOCK]**

In the past month, have you...

**PTSD2**. Had nightmares about the event(s) or thought about the event(s) when you did not want to?

1. Yes
2. No
3. Decline to answer

**PTSD3**. Tried hard not to think about the event(s) or went out of your way to avoid situations that reminded you of the event(s)?

1. Yes
2. No
3. Decline to answer

**PTSD4**. Been constantly on guard, watchful, or easily startled?

1. Yes
2. No
3. Decline to answer

**PTSD5**. Felt numb or detached from people, activities, or your surroundings?

1. Yes
2. No
3. Decline to answer

**PTSD6**. Felt guilty or unable to stop blaming yourself or others for the event(s) or any problems the event(s) may have caused?

1. Yes
2. No
3. Decline to answer

## Everyday Experiences of Discrimination

**Discrimination1**. We would like to ask you about your experiences with discrimination. Some of these questions may be difficult to answer. You can take your time and you may choose not to answer. Have you ever experienced discrimination, been prevented from doing something, or been hassled or made to feel inferior, in any of the following six situations because of **your race or color**? **Please select all that apply.**

1. At school
2. Getting a job
3. At work
4. Getting housing
5. Getting medical care
6. From the police or the courts
7. None of the above **[EXCLUSIVE]**
8. Decline to answer **[EXCLUSIVE]**

**Discrimination2.** Have you ever experienced discrimination, been prevented from doing something, or been hassled or made to feel inferior, in any of the following six situations because of your **sexual orientation or identity**? **Please select all that apply.**

1. At school
2. Getting a job
3. At work
4. Getting housing
5. Getting medical care
6. From the police or the courts
7. None of the above **[EXCLUSIVE]**
8. Decline to answer **[EXCLUSIVE]**

**[DO NOT DISPLAY Discrimination3 IF Discrimination1 AND Discrimination2 = None of the above OR Decline to answer]**

| In response to the discrimination asked about above: If you feel you’ve been treated unfairly, how do you usually respond? Do you: | Yes | No | Decline to answer |
| --- | --- | --- | --- |
| **Discrimination3_1**. Accept it as a fact of life? |  |  |  |
| **Discrimination3_2.** Try to do something about it? |  |  |  |
| **Discrimination3_3.** Talk to other people about it? |  |  |  |
| **Discrimination3_4.** Keep it to yourself? |  |  |  |

## Brief Connor-Davidson Resilience Scale (CD-RISC-10)

| How much do you agree or disagree with the following statements? | Strongly Disagree | Disagree | Neither Agree or Disagree | Agree | Strongly Agree |
| --- | --- | --- | --- | --- | --- |
| **CDR1.** I am able to adapt when changes occur. |  |  |  |  |  |
| **CDR2.** I can deal with whatever comes my way. |  |  |  |  |  |
| **CDR3.** I try to see the humorous side of things when I am faced with problems. |  |  |  |  |  |
| **CDR4.** Having to cope with stress can make me stronger. |  |  |  |  |  |
| **CDR5.** I tend to bounce back after illness, injury or other hardships. |  |  |  |  |  |
| **CDR6.** I believe I can achieve my goals, even if there are obstacles. |  |  |  |  |  |
| **CDR7.** Under pressure, I stay focused and think clearly. |  |  |  |  |  |
| **CDR8.** I am not easily discouraged by failure. |  |  |  |  |  |
| **CDR9.** I think of myself as a strong person when dealing with life’s challenges and difficulties. |  |  |  |  |  |
| **CDR10.** I am able to handle unpleasant or painful feelings like sadness, fear, and anger. |  |  |  |  |  |

## Multidimensional Scale of Perceived Social Support

| How much do you agree with the following statements? | Very Strongly Disagree | Strongly Disagree | Mildly Disagree | Mildly Agree | Strongly Agree | Very Strongly Agree |
| --- | --- | --- | --- | --- | --- | --- |
| **PerSocSup1**. There is a special person who is around when I am in need. |  |  |  |  |  |  |
| **PerSocSup2**. There is a special person with whom I can share my joys and sorrows. |  |  |  |  |  |  |
| **PerSocSup3**. My family really tries to help me. |  |  |  |  |  |  |
| **PerSocSup4**. I get the emotional help and support I need from my family. |  |  |  |  |  |  |
| **PerSocSup5**. I have a special person who is a real source of comfort to me. |  |  |  |  |  |  |
| **PerSocSup6**. My friends really try to help me. |  |  |  |  |  |  |
| **PerSocSup7**. I can count on my friends when things go wrong. |  |  |  |  |  |  |
| **PerSocSup8**. I can talk about my problems with my family. |  |  |  |  |  |  |
| **PerSocSup9**. I have friends with whom I can share my joys and sorrows. |  |  |  |  |  |  |
| **PerSocSup10**. There is a special person in my life who cares about my feelings. |  |  |  |  |  |  |
| **PerSocSup11**. My family is willing to help me make decisions. |  |  |  |  |  |  |
| **PerSocSup12**. I can talk about my problems with my friends. |  |  |  |  |  |  |

## Childhood Sexual Abuse

**ChildForce**. When you were age 16 or younger, was there ever a time when you were forced or frightened by another person to do something sexual with them when you didn’t want to?

1. Yes
2. No
3. Decline to answer

**[DISPLAY FOLLOWING IF ChildForce = YES]**

**ForcePartAge.** The first time that this happened, how old was this partner?

1. More than 5 years younger than I was
2. Around the same age as I was
3. 5-9 years older than me
4. More than 10 older than me
5. Not sure
6. Decline to answer

**AgeFirstTime.** How old were you the first time this happened?

1. [Number entry validated to < 16]
2. Decline to answer

**ManyTimes**. How many times did this happen before you turned 17?

1. [Number entry validated to < 999]
2. Decline to answer

**MorePersons**. Did this ever happen with more than one person?

1. Yes
2. No
3. Decline to answer

## Intimate Partner Violence

| Intimate relationships are characterized by many different feelings and behaviors. Sometimes relationships involve unwanted physical or emotional violence.  In the **past five years** has a romantic partner (e.g, boyfriend, girlfriend, etc)... | Yes | No | Decline to answer |
| --- | --- | --- | --- |
| **PartVioRec1.** Hit you with fists or an open hand? |  |  |  |
| **PartVioRec2.** Thrown something at you? |  |  |  |
| **PartVioRec3.** Verbally threatened you in any way? |  |  |  |
| **PartVioRec4.** Verbally demeaned you in front of strangers? |  |  |  |
| **PartVioRec5.** Pushed or shoved you? |  |  |  |
| **PartVioRec6.** Forced you to get high or drunk? |  |  |  |
| **PartVioRec7.** Made fun of your appearance? |  |  |  |
| **PartVioRec8.** Forced you to have sex? |  |  |  |
| **PartVioRec9.** Kicked you? |  |  |  |
| **PartVioRec10.** Hit you with an object? |  |  |  |
| **PartVioRec11.** Damaged or destroyed your property? |  |  |  |
| **PartVioRec12.** Stalked you? |  |  |  |

## Substance Use Treatment

| In the **past year**, have you… | Yes | No | Decline to Answer |
| --- | --- | --- | --- |
| **SUtreat1**. Been admitted into a hospital room to detox from drugs or alcohol? |  |  |  |
| **SUtreat2**. Have you been admitted to the hospital for any reason other than detoxification from drugs or alcohol? |  |  |  |
| **SUtreat3**. Have you visited an emergency room and not been admitted to the hospital? |  |  |  |
| **SUtreat4**. Spent one or more nights in a residential alcohol or drug treatment facility? |  |  |  |
| **SUtreat5.** Attended any one-on-one or group visits with a substance use disorder counselor? |  |  |  |
| **SUtreat6**. Received any outpatient substance use disorder treatment? |  |  |  |
| **SUtreat7.** Attended any 12-step meetings (for example AA, NA, CMA)? |  |  |  |
| **SUtreat8**. Thought about talking to a medical or mental health provider about your drug or alcohol use? |  |  |  |
| **SUtreat9**. Actually talked to a medical or mental health provider about your drug or alcohol use? |  |  |  |
| **SUtreat10**. Been to an emergency room due to alcohol or drugs? |  |  |  |
| **SUtreat11**. Attempted to hide (or lied about) your drug or alcohol use from others? |  |  |  |
| **SUtreat12**. Taken large enough quantities of a drug or alcohol to the point that you were at risk of injury or death? |  |  |  |
| **SUtreat13**. Received medication to treat an alcohol use disorder? |  |  |  |
| **SUtreat14**. Received medication to treat another type of mental illness? |  |  |  |
| **SUtreat15**. Outside of the services mentioned above, talked to a therapist about problems with your alcohol or drug use? |  |  |  |

**[DISPLAY 5a and 5b if SUtreat7 = YES]**

**SUtreat5a.** Do you have a sponsor?

1. Yes
2. No

**SUtreat5b**. Are you serving as a sponsor?

1. Yes
2. No

**[DISPLAY GHB1-3 IF ASSIST1_7 = YES] CHECK THIS NUMBER FOR ACCURACY IN REVISED ASSIST**

**GHB1**. Have you ever taken so much GHB that you fell asleep when you were not intending to?

1. Yes
2. No
3. Decline to answer

**GHB1a**. How many times has this happened?

1. 1 time
2. 2 times
3. 3 times
4. 4 times
5. 5+ times

**GHB2**. Have you taken so much GHB that you lost consciousness?

1. Yes
2. No
3. Decline to answer

**GHB2a**. How many times has this happened?

1. 1 time
2. 2 times
3. 3 times
4. 4 times
5. 5+ times

**GHB3**. Have you overdosed on GHB?

1. Yes
2. No
3. Decline to answer

[**Display GHB3a if GHB3=A]**

**GHB3a**. How many times has this happened?

1. 1 time
2. 2 times
3. 3 times
4. 4 times
5. 5+ times

**[DISPLAY GHB1 & GHB2 IF ASSIST1_8=YES]**

**GHB1**. In the **last year**, have you taken so much G you fell asleep without intending to (e.g., g'd out, or g sleeping)?

1. Never
2. Once
3. Twice
4. Three
5. Four
6. Five or more

**GHB2**. In the **last year**, have you overdosed on G (e.g., shaking, seizures, vomiting, unconscious and unrousable)?

1. Never
2. Once
3. Twice
4. Three
5. Four
6. Five or more

**[DISPLAY AmphetScared IF ASSISTrev1_2 or ASSISTrev1_3 ASSISTrev1_4 ASSISTrev1_5 = YES]**

**AmphetScared**. Has your meth, crack, coke or other uppers use caused you to feel sick or really scared (like your life may be in danger).

1. Yes
2. No
3. Decline to answer

**[DISPLAY MethTreat and MethPresc IF ASSISTrev1_5 = YES]**

**MethTreat.** Have you been treated for your meth use with mirtazapine (Remeron), bupropion (Wellbutrin), or injectable naltrexone (Vivitrol)?

1. Yes
2. No
3. I don’t know
4. Decline to answer

**MethPresc.** Have you used prescription stimulants (whether prescribed to you or not) such as Adderall or Ritalin to manage your meth use (or in place of meth)?

1. Yes
2. No
3. I don’t know
4. Decline to answer

**DiagADHD**. Have you been diagnosed with ADHD?

1. Yes
2. No
3. I don’t know
4. Decline to answer

**[DISPLAY ThinkADHD IF DiagADHD = No or I don’t know]**

**ThinkADHD.** Do you think you may have ADHD?

1. Yes
2. No
3. Decline to answer

**SelfPerAlcTreat.** In the past year, did you ever think you needed treatment for your alcohol use?

1. Yes
2. No
3. Decline to answer

**SelfPerDruTreat**. In the past year, did you ever think you needed treatment for your drug use?

1. Yes
2. No
3. Decline to answer

**OpioidMed**. In the past year, have you received medication to treat opioid use disorder?

1. Yes
2. No
3. I don’t know
4. Decline to answer

**[IF OpioidMed = Yes DISPLAY OpMedType]**

**OpMedType**. Which medications did you receive? Please select all that apply.

1. Buprenorphine (oral)
2. Buprenorphine (implant or injection)
3. Naltrexone (oral)
4. Naltrexone (injection)
5. Methadone
6. Something else (Please specify): ________
7. I don’t know

**MedVisits**. In the past year, how many times have you visited a medical office to see a healthcare provider?

1. Never
2. Once
3. Twice
4. 3-4 times
5. 5-6 times
6. 7-12 times
7. 13 or more times

## Acculturation

**BA1.** What language(s) do you speak at home?

1. Only English
2. English more than some other language
3. English and some other language equally
4. Some other language more than English
5. Only some other language

**BA2.** What language(s) do you speak with your friends?

1. Only English
2. English more than some other language
3. English and some other language equally
4. Some other language more than English
5. Only some other language

**BA3.** What language(s) do you read in?

1. Only English
2. English more than some other language
3. English and some other language equally
4. Some other language more than English
5. Only some other language

**BA4.** What language(s) do you think in?

1. Only English
2. English more than some other language
3. English and some other language equally
4. Some other language more than English
5. Only some other language

##

## Douching

Many people **douche** by using an enema, shower attachment (shower shot), anal douche bulb or other device to clean out their butt before or after receptive anal sex (when they bottom). We are interested in understanding when and how you may have douched in the past 3 months.

**Douche1**. **During the past 3 months**, have you ever douched to clean out your butt?

1. Yes
2. No

**[Skip to End of block if Douche1=B]**

**[DISPLAY Douche1a if Douche1 = YES]**

**Douche1a.** When was the last time you douched?

1. In the past day
2. 1-3 days ago
3. 4-10 days ago
4. 11-30 days ago
5. 30 or more days

**Douche2**. During the past 3 months, what device(s) did you use to douche? Choose all that apply:

1. An enema bottle or rubber bulb
2. A hose attached to the shower or a faucet
3. Something else (Please describe):

**Douche3**. During the past 3 months, what type of liquid did you use to douche? Choose all that apply:

1. Tap water only
2. Tap water and soap
3. A commercial douche or enema (i.e. Fleet)
4. Something else (Please describe):

**[DISPLAY Douche3a IF Douche3 = C]**

**Douche3a**. Please provide the names of the products(s) you usually buy to douche:

1. [Text entry]

**Douche4**. In general, how long does it take you to douche?

1. Less than 10 minutes
2. 10 to 30 minutes
3. Between 30 minutes and an hour
4. More than an hour

**Douche6**. During the **past 3 months,** have you had any bleeding from your anus after you douched?

1. Yes
2. No

**Douche7**. During the **past 3 months**, have you taken any fiber supplements (Fibercon, Pure) to make it easier to clean out your butt when you douche?

1. Yes
2. No

**Douche8**. During the **past 3 months**, how many days have you douched?

1. 0-90+

**Douche9**. During the **past 3 months**, how many days have you douched to clean out your butt **before** receptive anal sex (you were the bottom)?

1. 0-90+

**Douche10**. During the **past 3 months**, how many days have you douched to clean out your butt **after** receptive anal sex (you were the bottom)?

1. 0-90+

## Contact Information

In order for us to send you your test kits and pay you we need to know who you are and how to reach you.

A**. FirstName**. [Text box]

1. **LastName**. [Text box]

**ConfirmEmail**

Here is the email and phone number you gave us when you joined the study. Please check the box next to any information that needs to be updated.

1. Preferred Email: [EMBEDDED EMAIL FROM SCREENER]
2. Preferred Phone: [ EMBEDDED PHONE FROM SCREENER]
3. All my contact information is correct [exclusive]

**[DISPLAY EMAILUPDATE IF ConfrimEmail is ‘Preferred Email’]**

**EmailUpdate**

Please enter your updated email address below.

[TEXTBOX]

**[DISPLAY EMAILUPDATE IF ConfrimEmail is ‘Preferred Phone]**

**PhoneUpdate**Please enter your updated phone number below

[TEXTBOX]

**MailingAddress.** To participate in the study we need to be able to send you your testing kit materials. What address would you like us to send them to?

1. Address1:
2. Address2:
3. City:

**State**: [Dropdown of US States]

**Zip**: [validated to US Postal codes]

**[Display OtherState if State=OTHER]**

**OtherState:** [TEXT Entry of Other US Territory or area name]

**TEXTOk:** Is it okay to text?

1. Yes
2. No

**MsgOk:** Is it okay to leave messages?

1. Yes
2. No

**[PHONE NUMBER CONFIRMATION DIALOGUE APPEARS ON NEXT PAGE]**
