# Supplementary material for: The American Transformative HIV Study: Protocol for a US National Cohort of Sexual and Gender Minority Individuals With HIV
Source: JMIR Public Health Surveill. 2025 May 22;11:e66921. doi: 10.2196/66921 (PMC12121540; doi:10.2196/66921)
Supplement: Multimedia Appendix 3 [file publichealth-v11-e66921-s003.docx]

AMETHST Screening Survey

Contents

[Now or Later 2](#_Toc107235086)

[Age and Juvenile Comprehension Assessment 3](#_Toc107235087)

[Demographics 4](#_Toc107235088)

[HIV, PrEP, and PEP Status 5](#_Toc107235089)

[Sexual Behavior 8](#_Toc107235090)

[10 Item Personality Inventory 9](#_Toc107235091)

[Drug Use 10](#_Toc107235092)

[STIs 12](#_Toc107235093)

[Monkeypox 13](#_Toc107235094)

[Contact Information 14](#_Toc107235095)

## Now or Later

**NowOrLater**. This survey will take approximately 5 minutes to complete. We can also email you a link to the survey so you can complete it at a later time.

1. I want to complete the survey now.
2. I will complete the survey later. Please email me a link.

**EmailForLater**. Email address:

1. [text entry box]

## Age and Juvenile Comprehension Assessment

**Age**. How old are you?

1. [Number entry validated to < 99]

**[IF Age ≤ 15, SKIP TO INELIGIBLE FULL STOP, IF > 49 ALLOW TO FINISH SURVEY BUT END ON INELIGIBLE ASK FOR CONTACT FOR FUTURE RESEARCH]**

**[IF Age < 18, DISPLAY CONSENT1, CONSENT2, CONSENT3]**

**Consent1**. What is this study focused on?

1. Sexuality, sexual health, and substance use
2. Eating habits
3. Sleeping patterns

**Consent2**. Which of these is a risk of the study?

1. Side effects from a treatment being used
2. Exercise related fatigue
3. Discomfort answering personal questions

**Consent3**. How long is this survey expected to take?

1. About 5 minutes
2. About 30 minutes
3. About 60 minutes

## Demographics

**EthnExpand.** Which racial or ethnic group do you belong to? **Please select all that apply.**

1. Black or African American
2. East Asian
3. Latino, Latinx, or Latin American
4. Middle Eastern or North African
5. Native American, American Indian, or Alaska Native
6. Native Hawaiian or other Pacific Islander
7. South Asian
8. White or European American
9. Multiracial or Multiethnic
10. Something else (Please specify):

**Cis (cisgender) refers to someone who currently identifies with the sex they were assigned at birth.**

**Trans (transgender) refers to someone who currently identifies differently than the sex they were assigned at birth**

**Gender.** What gender do you currently identify as?

1. Cis man
2. Cis woman
3. Trans man
4. Trans woman
5. Non-binary, genderqueer, genderfluid
6. Agender
7. Questioning
8. Something else (Please specify):

**SexBirth.** What sex were you assigned at birth?

1. Male
2. Female

**SexOrientation.** Which best describes how you identify your sexual orientation?

1. Gay (homosexual)
2. Bisexual
3. Straight (heterosexual)
4. Queer
5. Pansexual
6. Sexually fluid
7. Something else (Please specify): ________

**USAYN**. Do you live in the US or one of its territories?

1. Yes
2. No

## HIV, PrEP, and PEP Status

**HIVStatus**. What is your HIV status?

1. HIV-negative
2. HIV-positive
3. I don’t know; I am unsure

**[DISPLAY WhenDiag IF HIVStatus = positive]**

**WhenDiag**. How long ago was your HIV diagnosis?

1. Less than 3 months ago?
2. 3-6 months ago
3. 6-12 months ago
4. 1-2 years ago
5. 2-5 years ago
6. 5-10 years ago
7. 10+ years ago.

**PrEPstatus**. Have you ever been prescribed PrEP (Pre-Exposure Prophylaxis) to prevent HIV (e.g. Truvada/Descovy/etc.)?

1. Yes, but I am not currently taking PrEP
2. Yes, I am currently on PrEP
3. No, never taken PrEP
4. I don’t know what PrEP is

**PEPstatus**. Have you ever been prescribed HIV medications **AFTER an exposure** to prevent getting HIV (for example, sex without a condom or the condom broke). This is called **PEP (Post Exposure Prophylaxis).**

1. Yes, in the last year
2. Yes, more than one year ago
3. No, never

**ClinicTrial**. Are you currently participating in an HIV vaccine or HIV drug prevention clinical trial?

1. Yes
2. No

**[IF PrEPstatus = never or don’t know SKIP TO END OF SECTION]**

**PrEPdenial.** Has your insurance **ever** denied covering your PrEP prescription? **Please select all that apply.**

1. Yes, for Truvada
2. Yes, for emtricitabine/tenofovir (generic Truvada)
3. Yes, for Descovy
4. Yes, for injectable PrEP (i.e., cabotegravir/Apretude)
5. No
6. Not applicable

**[DISPLAY PrePdenial 2 and 3 IF PrEPdenial = any yes]**

**PrEPdenial2**. How many times has this happened?

1. [NUMBER ENTRY]

**PrEPdenial3**. What year did the **most recent** denial occur?

1. [VALIDATED YEAR ENTRY]

**OOPrEP. Within the past two years,** have you experienced any “out of pocket” medical expenses for PrEP? “Out of pocket” refers to **any** amount of money you have to pay toward a medical service that is NOT covered by any insurance or special assistance you might have. **Please select all that apply.**

- 1. Yes, for my prescription
  2. Yes, for check ups
  3. Yes, for lab work
  4. Yes, something else
  5. No [EXCLUSIVE]
  6. Not sure/Not applicable [EXCLUSIVE]

**[IF OOPrEP = any yes DISPLAY OOPrEPinsurance1 and OOPrEPinsurance2]**

**OOPrEPinsurance1.** What type of insurance did you have at the time of these “out of pocket” expenses?

- 1. Private insurance through my employer, union or school
  2. Private insurance through the individual Marketplace
  3. Medicaid
  4. Medicare
  5. Something else (Please specify): ________
  6. I did not have insurance
  7. Not sure/Don’t remember

**OOPrEPinsurance2**. What insurance provider and plan did you have at the time of these “out of pocket” expenses? For example: “United Health, PPO plan.” Please type whatever information you know, or select "Don't know."

1. [TEXT ENTRY]
2. Don’t know/Don’t remember

## Sexual Behavior

**MalePartners6m**. In the last **6 months**, how many **cis men (i.e. not trans men)** have you had **anal** sex with?

1. [number entry validated to ?]

**FemalePartners6m**. In the last **6 months**, how many **cis women (i.e. not trans women)** have you had **anal/vaginal** sex with?

1. [number entry validated to ?]

**TWPartners6m**. In the last **6 months**, how many **trans women** have you had **anal/vaginal** sex with?

1. [number entry validated to ?]

**TMPartners6m.** In the last **6 months**, how many **trans men** have you had **anal/vaginal/front hole** sex with?

1. [number entry validated to ?]

**URAIcis6m**. In the last **6 months**, how many times did you have **receptive anal sex** (you were the bottom) with a **cis man without a condom**?

1. [number entry validated to ?]

**[DISPLAY IF TWPartners6m >0]**

**URAItw6m.** In the last 6 months, how many times did you have **receptive anal sex** (you were the bottom) with a **trans woman without a condom?**

1. [number entry validated to ?]

**UIAIcis6m.** In the last **6 months**, how many times did you have **insertive anal sex** (you were the top) with a **cis man without a condom**?

1. [number entry validated to ?]

**[DISPLAY IF TWPartners6m >0]**

**UIAItw6m.** In the last **6 months**, how many times did you have **insertive anal/vaginal sex** (you were the top) with a **trans woman without a condom**?

1. [number entry validated to ?]

**[DISPLAY IF TMPartners6m >0]**

**UIAItm6m.** In the last **6 months**, how many times did you have **insertive anal /vaginal/front hole sex** (you were the top) with a **trans man without a condom**?

1. [number entry validated to ?]

**UAVIFemale6m**. In the last **6 months**, how many times did you have **anal/vaginal sex** with a **cis woman without a condom**?

1. [number entry validated to ?]

**RecentSexTotal**. In the last **two weeks**, how many people have you had sex with?

1. [NUMBER ENTRY VALIDATED <~200}

**RecentSexGender**. Of these sex partners, what were their genders? **Please select all that apply.**

1. Cis man
2. Cis woman
3. Trans man
4. Trans woman
5. Non-binary, genderqueer, genderfluid
6. Agender
7. Questioning
8. Something else (Please specify):

**RecentSexMain**. In the last **two weeks,** how many people have you had sex with who were your **main partner** in a committed relationship?

1. [NUMBER ENTRY VALIDATED <10]

**RecentSexCasual**. In the last **two weeks**, how many people have you had sex with who were **casual but repeated** sex partners (i.e. a “fuck buddy”)?

1. [NUMBER ENTRY VALIDATED <~100]

**RecentSexNew**. In the last **two weeks**, how many **new** sex partners have you had (i.e., someone you haven’t had sex with before)?

1. [NUMBER ENTRY VALIDATED <~200]

**ForceEncounter.** In the past **five years**, have you had a sexual encounter that you did not consent to? **Please select all that apply.**

A. No [EXCLUSIVE]

B. Yes, while under the influence of drugs or alcohol

C. Yes, not under the influence of drugs or alcohol

D. Decline to answer. [EXCLUSIVE]

## Drug Use

**Pot3Mdays.** In the last **3 months**, about how many days have you used marijuana (i.e. pot, cannabis, weed, etc.)?

1. I have not used in the last three months
2. 1-5 days
3. 6-10 days
4. 11-20 days
5. 21-30 days
6. 30+ days

**Meth3Mdays.** In the last **3 months**, about how many days have you used methamphetamine (i.e. crystal meth, tina, ice, etc.)?

1. I have not used in the last three months
2. 1-5 days
3. 6-10 days
4. 11-20 days
5. 21-30 days
6. 30+ days

**Cocaine3Mdays.** In the last **3 months**, about how many days have you used cocaine or crack (i.e. coke, blow, crack cocaine, rock, free base cocaine)?

1. I have not used in the last three months
2. 1-5 days
3. 6-10 days
4. 11-20 days
5. 21-30 days
6. 30+ days

**Inject**. In the **last year**, have you **injected** any drugs recreationally?

1. Yes
2. No

**[DISPLAY InjectWhat IF Inject = YES]**

**InjectWhat**. In the **last year**, what drugs have you **injected** recreationally?

1. Methamphetamine (crystal meth, tina, ice, etc.)
2. Cocaine or crack (coke, blow, crack, crack cocaine, etc.)
3. Street opioids (heroin, opium, etc.)
4. Prescription opioids (morphine, codeine, fentanyl, oxycodone/OxyContin/Percocet, hydrocodone/Vicodin, methadone, buprenorphine/Suboxone, etc.)
5. Ketamine (k, special k)
6. Something else (Please specify):

## STIs

| The following is a list of sexually transmitted infections. For each infection, please indicate when or if you were diagnosed | In the last 6 Months | 6-12 Months | 1-5 Years | More than 5 years | Not applicable |
| --- | --- | --- | --- | --- | --- |
| **STI1.** Gonorrhea or Chlamydia - Rectal/Anal |  |  |  |  |  |
| **STI2.** Gonorrhea or Chlamydia - Oral/Pharyngeal |  |  |  |  |  |
| **STI3.** Gonorrhea or Chlamydia - Urethral |  |  |  |  |  |
| **STI4.** Genital or anal warts, HPV |  |  |  |  |  |
| **STI5.** Genital herpes, HSV1, HSV2 (diagnosis or outbreak) |  |  |  |  |  |
| **STI6.** Syphilis |  |  |  |  |  |
| **STI7.** Hepatitis A |  |  |  |  |  |
| **STI8.** Hepatitis B |  |  |  |  |  |
| **STI9.** Hepatitis C |  |  |  |  |  |
| **STI10.** Urethritis |  |  |  |  |  |

**AntibioSTI.** In the last year, have you taken antibiotics before or after sex to prevent (i.e., before you get diagnosed or have symptoms) getting a STI like chlamydia or syphilis?

1. Yes, after having sex
2. Yes, before sex
3. Yes, before and after sex
4. No, I haven’t done this or have only taken antibiotics **after being diagnosed with an STI**
5. I don’t know

## Monkeypox *(Please note at the time this survey was in use, mpox was called “monkeypox”)*

Monkeypox is a disease caused by a virus like smallpox. It can be transmitted through sex via bodily fluids or close contact to sores (pox lesions). Current evidence also indicates that rates of infection may be higher among gay, bisexual, and other men who have sex with men.

**MPXworry**. Over the last **two weeks,** how many days have you worried about getting monkeypox?

1. Never
2. One or two days
3. Some days
4. More than half the days
5. Nearly every day
6. Every day
7. I have already had monkeypox

**MPXlikely.** The Centers for Disease Control and Prevention (CDC) recommends that people who may be exposed to monkeypox get vaccinated with smallpox vaccines that are currently available. If you were offered smallpox vaccine, how likely would you be to take it?

1. Extremely unlikely
2. Unlikely
3. Neutral
4. Likely
5. Extremely likely
6. I have already been vaccinated for smallpox

**MPXlikelynew.** The Centers for Disease Control and Prevention (CDC) recommends that people who may be exposed to monkeypox get vaccinated with smallpox vaccines that are currently available. If you were offered smallpox vaccine, how likely would you be to take it?

1. Extremely unlikely
2. Unlikely
3. Neutral
4. Likely
5. Extremely likely
6. I have already received at least one dose of the monkeypox/smallpox vaccine

**MPXtried.** Since May 2022, have you tried to get the monkeypox (smallpox) vaccine?

1. Not applicable, I was vaccinated for monkeypox (smallpox) before May 2022
2. Yes, and I was able to get vaccinated
3. Yes, but I was **not** able to vaccinated
4. No, I have not tried to get vaccinated

**[DISPLAY MPXvaccdoses if MPXtried=B]**

**MPXvaccdoses.** How many doses of the monkeypox (smallpox) vaccine have you received?

1. 1
2. 2

**MPXvaccbehaviors.** Since receiving at least one dose of the monkeypox (smallpox vaccine), have you changed your sexual behavior?

|  | Decreased a lot | Decreased some | No change | Increased some | Increased a lot |
| --- | --- | --- | --- | --- | --- |
| Number of sex partners |  |  |  |  |  |
| Number of strangers I have sex with (anonymous hookups) |  |  |  |  |  |
| Number of sex acts (e.g., oral, anal, kissing, topping, bottoming) |  |  |  |  |  |
| Asked sex partners if they received a monkeypox vaccine |  |  |  |  |  |
| Condom use |  |  |  |  |  |
| Visits to places and events like bathhouses, sex parties, etc. |  |  |  |  |  |
| Seeking out sex partners that have already recovered from monkeypox |  |  |  |  |  |

**MPXavail.** As far as I know, there is a place near me where I could get the monkeypox (smallpox) vaccine if I wanted to.

1. Agree
2. Disagree
3. I don’t know

**MPXavoid.** Since June 2022, have you avoided places where you are concerned you might get monkeypox? For example places where people will be partially or fully undressed like clubs, circuit parties, sex parties, etc..

1. Yes
2. No
3. Not applicable

**MPXsex.** Since May 2022, have you changed your sexual behavior because you were concerned about monkeypox? **Please select all that apply.**

1. No I haven't made any changes [EXCLUSIVE]
2. Reduced my number of sex partners
3. Tried to only have sex with people I know
4. Engaged in fewer sex acts
5. Asked sex partners if they have monkeypox symptoms
6. Used condoms
7. Stopped or reduced attendance at places like bathhouses, sex parties, etc
8. Sought out sex partners that have already recovered from monkeypox
9. Other (Please specify): ____

**MPXsexRecent.** **In the last 30 days**, have you changed your sexual behavior because you were concerned about monkeypox? **Please select all that apply.**

1. No I haven't made any changes [EXCLUSIVE]
2. Reduced my number of sex partners
3. Tried to only have sex with people I know
4. Engaged in fewer sex acts
5. Asked sex partners if they have monkeypox symptoms
6. Used condoms
7. Stopped or reduced attendance at places like bathhouses, sex parties, etc
8. Sought out sex partners that have already recovered from monkeypox
9. Other (Please specify): ____

**MPXexpose.** Since May 2022, have you been told that you may have been exposed to monkeypox? Please select all that apply.

1. Told by a sex partner that I may have been exposed
2. Told by a healthcare provider that I may have been exposed
3. Told by someone who was not a sex partner that they had monkeypox
4. Not applicable [EXCLUSIVE]

**MPXtest.** Since May 2022, have you sought testing for monkeypox?

1. Yes, and I received a test
2. Yes, but I was not able to test
3. No

**[IF MPXtest = A DISPLAY MPXtest2]**

**MPXtest2.** Did you receive an out-of-pocket bill for your monkey pox test?

1. No
2. Yes, between $10 and $49
3. Yes, between $50 and $79
4. Yes, between $80 and $100
5. Yes, more than $100

**MPXDxNew.** Since May 2022, have you had monkeypox?

1. Yes, I was diagnosed with monkeypox
2. Yes, I think I had monkeypox, but was never diagnosed
3. No
4. I don’t know

**[IF MPXDx= C or D SKIP TO END OF BLOCK**

**[DISPLAY MPXDxDate IF MPXDxNew=A]**

**MPXDxDate.** On what date were you diagnosed with monkey pox?

1. Year [FORM ENTRY]
2. Month [FORM ENTRY]
3. Day (optional) [FORM ENTRY]

**[DISPLAY MPXthinkDate IF MPXDxNew=B]**

**MPXthinkDate.** On what date, do you think you developed of monkeypox?

1. Year [FORM ENTRY]
2. Month [FORM ENTRY]
3. Day (optional) [FORM ENTRY]

**MPXDx.** Since May 2022, have you had monkeypox?

1. Yes
2. No
3. I don’t know

**[IF MPXDx = B or C SKIP TO END OF SECTION]**

**MPXTreat1.** Did you seek treatment for monkeypox?

1. Yes, and I received treatment
2. Yes, but my doctor decided I **did not need** treatment
3. Yes, but my doctor **could not** provide me treatment
4. No

**[IF** **MPXTreat1. = A DISPLAY MPXTreat2]**

**MPXTreat2.** What type of treatment did you receive? **Please select all that apply.**

1. TPOXX
2. Prescription strength painkillers
3. Something else (Please specify):____________

**MPXExp.** Please rate your pain experience during the worst of your monkeypox infection?

1. No pain
2. Mild pain
3. Moderate pain
4. Severe pain
5. Very severe pain

**MPXwork.** Did you miss work because of having monkeypox?

1. Yes
2. No
3. Not applicable

**[IF MPXwork. = A DISPLAY MPXwork2]**

**MPXwork2.** How many days of work did you miss?

1. 1-3
2. 4-6
3. 7-10
4. 11-14
5. 15-19
6. 20 or more days

**MPXpay.** As a result of monkeypox, have you experienced any of the following?**Please select all that apply.**

1. Difficulty paying for food
2. Difficulty paying your rent or mortgage
3. Difficulty paying for other essential bills (electric, internet, medical bills, etc.)
4. Difficulty covering other basic necessities (Please specify):__________
5. None of the above [EXCLUSIVE]

## Ending Eligible

Thank you! It looks like you may be eligible to participate in Together 5,000. We want you to get to know a little bit about who we are and why we are doing this study, so please watch this brief video.

[VIDEO HERE]

To participate in the study we will need to gather some contact information from you.

We will never share your contact information with anyone else and will only use that information to contact you regarding our research.

**MailOk**. Can you receive free at-home HIV test kits at your home (or a convenient alternative address) and return them to us? This is required for participation in the study.

1. Yes
2. No
3. I’m not sure, I have some questions

**[DISPLAY MailConfirm IF MailOK=C]**

**MailConfirm.** Thank you for your interest in AMETHST 5000.

However, to participate in the study, we need your contact information and an address to send test kits to. This address does not need to be your home, but can be a friend, partner or family member's home–or anywhere you feel comfortable receiving an HIV test kit. Our packages will never say anything descriptive (e.g., "HIV Test Kit") on the outside and will come in plain packaging with only your preferred name, address and a return address on the label.

If you would like to learn more about our mailing or testing process, please reach out to us! We would be happy to hear from you: [AMETHST5000@sph.cuny.edu](mailto:AMETHST5000@sph.cuny.edu)

Are willing to receive test kits to an address of your choosing?

1. Yes, I am willing to receive packages [ROUTE TO ELIGIBLE ENDING]
2. No, I am not willing to receive packages [ROUTE TO INELIGIBLE ENDING]
3. Please contact me to discuss my options [ROUTE TO INELIGIBL ENDING]

## Ending Ineligible

Thank you for taking the time to complete our survey. It doesn’t look like you are eligible at the present time, but we can reach out to you should our eligibility criteria change or if we have another study that you might be interested in. We will never share your contact information with anyone outside of the study.

## Contact Information

**Email**. What is your email address?

1. [email entry]

**EmailVerify**. [email entry]

To communicate with you about the study or future studies, what is your phone number? Your information is kept private and we will never spam you. You may choose not to provide a phone number now, but may be required to do so in the future to participate and receive a payment.

**Phone**. What is your phone number?

1. [phone entry]

**PhoneVerify.** [phone entry]

**FutureStudies.** Would you be willing to be contacted to participate in a future research study, including paid opportunities? We will never share your contact information with anyone else.

1. Yes
2. No
